# A peer-volunteer led active ageing programme to prevent decline in physical function in older people at risk of mobility disability (Active, Connected, Engaged [ACE]): study protocol for a randomised controlled trial

**DOI:** 10.1186/s13063-023-07758-3

**Published:** 2023-11-29

**Authors:** Afroditi Stathi, Janet Withall, Diane Crone, Helen Hawley-Hague, Rebecca Playle, Emma Frew, Sally Fenton, Melvyn Hillsdon, Christopher Pugh, Chris Todd, Kate Jolly, Nick Cavill, Max Western, Sarah Roche, Nigel Kirby, Elisabeth Boulton, Janice Thompson, Katie Chatwin, Amy Davies, Zsofia Szekeres, Colin Greaves

**Affiliations:** 1 School of Sport, Exercise and Rehabilitation Sciences, University of Birmingham, Edgbaston, Birmingham, B15 2TT UK; 2 Centre for Health, Activity and Wellbeing Research, Cardiff Metropolitan University, Cyncoed Campus, Cyncoed Road, Cardiff, CF23 6XD UK; 3 School of Health Sciences, University of Manchester, Oxford Road, Manchester, M13 9PL UK; 4 National Institute for Health and Care Research, Applied Research Collaboration-Greater Manchester, School of Health Sciences, Faculty of Biology, Medicine and Health, The University of Manchester, Manchester, M13 9PL UK; 5 Centre for Trials Research, Cardiff University, Cardiff, CF14 4YS UK; 6 Institute of Applied Health Research, University of Birmingham, Edgbaston, Birmingham, B15 2TT UK; 7 Department of Public Health and Sports Science, University of Exeter, Exeter, EX1 2LU UK; 8 Manchester Academic Health Science Centre, Manchester, M13 9NQ UK; 9 Manchester Institute for Collaborative Research On Ageing, Manchester, M13 9PL UK; 10 Manchester University NHS Foundation Trust, Manchester, M13 9WL UK; 11 Cavill Associates Ltd, Manchester, UK; 12 Centre for Motivation and Health Behaviour Change, Department for Health, University of Bath, Bath, BA2 4SY UK; 13 Royal Voluntary Service, Stoke-On-Trent, UK; 14 Age UK, London, UK

**Keywords:** Physical activity, Disability prevention, Well-being, Peer-volunteers, Frailty, Mobility, Community, Randomised controlled trial

## Abstract

**Background:**

The Active Connected Engaged [ACE] study is a multi-centre, pragmatic, two-arm, parallel-group randomised controlled trial [RCT] with an internal pilot phase. The ACE study incorporates a multi-level mixed methods process evaluation including a systems mapping approach and an economic evaluation. ACE aims to test the effectiveness and cost-effectiveness of a peer-volunteer led active ageing intervention designed to support older adults at risk of mobility disability to become more physically and socially active within their communities and to reduce or reverse, the progression of functional limitations associated with ageing.

**Methods/design:**

Community-dwelling, older adults aged 65 years and older (*n* = 515), at risk of mobility disability due to reduced lower limb physical functioning (Short Physical Performance Battery (SPPB) score of 4–9 inclusive) will be recruited. Participants will be randomised to receive either a minimal control intervention or ACE, a 6-month programme underpinned by behaviour change theory, whereby peer volunteers are paired with participants and offer them individually tailored support to engage them in local physical and social activities to improve lower limb mobility and increase their physical activity. Outcome data will be collected at baseline, 6, 12 and 18 months. The primary outcome analysis (difference in SPPB score at 18 months) will be undertaken blinded to group allocation. Primary comparative analyses will be on an intention-to-treat (ITT) basis with due emphasis placed on confidence intervals.

**Discussion:**

ACE is the largest, pragmatic, community-based randomised controlled trial in the UK to target this high-risk segment of the older population by mobilising community resources (peer volunteers). A programme that can successfully engage this population in sufficient activity to improve strength, coordination, balance and social connections would have a major impact on sustaining health and independence.

ACE is also the first study of its kind to conduct a full economic and comprehensive process evaluation of this type of community-based intervention. If effective and cost-effective, the ACE intervention has strong potential to be implemented widely in the UK and elsewhere.

**Trial registration:**

ISRCTN, ISRCTN17660493. Registered on 30 September 2021.

Trial Sponsor: University of Birmingham, Contact: Dr Birgit Whitman, Head of Research Governance and Integrity; Email: researchgovernance@contacts.bham.ac.uk.

Protocol Version 5 22/07/22.

**Supplementary Information:**

The online version contains supplementary material available at 10.1186/s13063-023-07758-3.

## Background

During old age, people gradually transition from independence and adequate physical function to frailty and mobility disability [1, 2]. Mobility disability, defined as a reduced ability to walk or balance, increases rapidly with age and generates major societal challenges. These include costs for people with mobility limitations such as loss of independence, risk of falls and reduced quality of life; costs to friends/family who provide care, and increased health and social care costs [3, 4]. Among adults aged over 70, 38% are classed as frail or pre-frail (defined as scoring 9 or less on the Short Physical Performance Battery (SPPB)) [1, 5]. Frail or pre-frail older people have a substantially higher risk of major mobility disability (OR = 8.3 (95% CI: 3.3 to 20.7) [5] and mortality (HR 2.6 to 5.3) [6] compared with non-frail older adults.

Increasing physical activity can prevent or delay progression of frailty and mobility disability [7, 8]. However, there is a clear trend of declining physical activity over time in people aged over 65 [9]. Reasons for this include the lack of a companion to go out with, low confidence to engage with community initiatives, and perceived safety of engaging with activity [10]. A recent systematic review of reviews examined interventions that were efficacious or effective in increasing uptake of physical activity in older people (17 studies; *N* = 79,650) and reported barriers to and facilitators of active ageing (9 studies; *N* = 22,413). Access to role models, peer and community support, individual tailoring of interventions, making exercise enjoyable and sociable, and feelings of ownership of interventions were identified as key enablers of physical activity [11].

A 2019 National Institute of Health Research (NIHR) review of UK-based physical activity interventions for older people [12] identified only two volunteering programmes, a peer-led walking group feasibility trial [13] and our Active Connected Engaged [ACE] feasibility trial [14]. This review highlighted the need for more robust evidence for such approaches ensuring that they incorporate theories of behaviour change; focus on social enablers of exercise; and target people with poor lower limb strength and/or weaker social networks.

Interventions that can successfully increase levels of physical activity and enhance social connections, leading to the delay or prevention of functional decline would have substantial public health value.

## Methods

### Trial design

The study protocol is presented in accordance with the Standard Protocol Items: Recommendations for Interventional Trials (SPIRIT) guidelines (Additional file 1).

The ACE study is an individually randomised, parallel-group, single-blind RCT with an internal pilot phase, a multi-level (individual and systems level), mixed-methods process evaluation and an economic evaluation. Following identification and recruitment, 515 patients who meet the study inclusion criteria will be randomised to receive either the ACE intervention, delivered over a period of 6 months by peer volunteers, or a minimal control intervention. Participants will be individually randomised to the intervention and control arms in a 1:1 ratio, stratified by site, using a centralised web-based system run by the Cardiff Centre for Trials Research.

The primary objective of the ACE study is to assess the effectiveness of a peer-volunteer led active ageing intervention for reducing the progression of mobility-related functional limitations in older people, who are at high risk of transition from independence to mobility-related disability. Secondary objectives are to compare participants allocated to the ACE programme with the control group in terms of levels of physical activity, psychological functioning and well-being, health-related quality of life, capability, activities of daily living, cognition, social networks and reduced sedentary time, loneliness, pain, falls, fear of falling and utilisation of health and social care.

The internal pilot phase will assess the feasibility of recruitment methods (allowing for some refinement if needed) and confirm adequate participant retention rates in the study, prior to progressing with the main trial (Additional file 2). Primary and secondary outcome data will be collected at baseline, 6, and 18 months. Selected secondary outcomes will also be assessed at 12 months.

The ACE study will be conducted at four UK sites (West Midlands, Greater Manchester, South Wales and Bristol), allowing recruitment of a socio-economically and ethnically diverse sample. At the delivery site in Wales, the study will be known as ACTIF, with the qualifier ‘known in England as ACE’ on documentation. This is to avoid any confusion with the ACE (Adverse Childhood Experiences) programme in Wales [15].

### Study population

The eligibility criteria are intended to identify sedentary, community-dwelling, older people aged 65 and over with lower limb functional limitations (i.e. at risk of major mobility limitations) but who are still ambulatory, i.e. they can still walk. This will be measured using a battery of objective physical function tests (SPPB) to assess balance, walking speed and the ability to move from a sitting to a standing position. This generates a physical function score from 0 to 12. Older adults with scores of 4–9 (inclusive) out of 12 will be eligible to take part in ACE. This is based on data showing that older adults with SPPB scores of 9 or less have a substantially higher risk of major mobility disability 3 years later (OR = 8.3 (95% CI: 3.3 to 20.67) compared with those with a score of 12 [5, 8]. Inclusion and exclusion criteria for participants and peer volunteers are summarised in Table 1.

**Table 1:** Inclusion and exclusion criteria for the selection of participants and peer volunteers

	**Participants**
**Inclusion criteria**	• Adults aged 65 or older who are not in full-time employment • Being at risk of major mobility disability (score between 4 and 9 (inclusive) on the Short Physical Performance Battery (SPPB)^a^ • Planning to reside in the target area (West Midlands, Greater Manchester, South Wales or Bristol) for at least 18 months
**Exclusion criteria**	• Self-reported high level of mobility^b^ or SPPB score above 9 at baseline screening • Self-reported inability to walk across a room without a walking aid other than a walking stick, or using a wheelchair or Zimmer frame • Existing major mobility limitation (defined as SPPB of 3 or less, or unable to complete the 4-m walk component of SPPB) • Living in residential or nursing care • Inability to meet regularly with an ACE volunteer • A documented or patient-reported medical condition that would preclude participation, including: o Severe arthritis that prevents participation in physical activity; o Parkinson’s disease or diagnosed dementia o Any terminal illness o Lung disease requiring use of orally administered corticosteroids or supplemental oxygen o Severe kidney disease requiring dialysis o Severe heart disease or an implanted cardiac defibrillator o Cardiac arrest which required resuscitation o Receiving radiation therapy or chemotherapy treatment for cancer o Awaiting knee or hip surgery o Major heart surgery in the last 6 months o Unstable heart condition (e.g. uncontrolled arrhythmia, angina, heart failure or hypertension) o Spinal surgery in the last 6 months o Any other clinical condition that the person’s GP or clinician considers would make them unsuitable for participation in an exercise programme
**Temporary exclusion criteria**	• Heart attack (or myocardial infarction), stroke, spinal surgery, hip fracture, hip or knee replacement within the previous 6 months • Currently receiving physical therapy on legs or enrolled in another physical activity research or intervention study
	**Peer volunteers**
**Inclusion criteria**	• Community-dwelling • Aged 55 years and older • SPPB scores of 4 and above • Not in full-time employment (unless with flexibility to volunteer during weekdays)
**Exclusion criteria**	• Not available to volunteer in the daytime during the week

^a^ Based on definitions of physical frailty from the European Medicines Agency, this guidance defines pre-frailty as an SPPB score of 8–9 and frailty as an SPPB score of 7 or less. ^b^ At screening reported that all of the following tasks are ‘easy to perform’: walking across a room, getting out of a low chair, walking up a flight of stairs with no handrail or wall to lean on and walking on uneven pavement without losing balance

### Recruitment

A range of recruitment strategies to identify suitable participants and peer-volunteers will be employed:

#### Primary care

General practitioner (GP) practices in the study catchment areas will be invited to participate through their local Clinical Research Network (CRN) and other networks. Where possible we will select practices to maximise diversity in terms of ethnicity and socio-economic status.

Practice staff will search the practices’ electronic patient databases for potentially eligible patients using the trial entry criteria (for criteria that are coded in the database). Lists generated from the searches will be reviewed by a GP to screen for items that are not included in the electronic searches. A Patient Approach Letter (PAL) printed on the practice headed notepaper, a reply form, Participant Information Sheet (PIS), and one-page study summary will be sent to suitable patients, with a pre-paid reply envelope addressed to the research team at the local trial site. The reply form will allow people to indicate whether they are interested in being a study participant or a peer-volunteer. GPs and practice nurses may also offer the recruitment pack in surgery to suitable patients.

#### Third sector organisations

The Principal Investigators at each trial site will engage with Voluntary, Community or Social Enterprise (VCSE) organisations and authorities working with adults over the age of 65 to identify potentially eligible service users. Publicity materials will be made available through libraries, supermarkets, post offices and GP surgeries.

#### Word-of-mouth and snowball sampling

To enhance recruitment, we will use word-of-mouth and snowball sampling techniques and employ the assistance of bi-lingual community champions.

#### Local media

Recruitment will be supported by a low-cost public relations campaign targeting local radio and community events.

Each ACE trial site will track recruitment methods to determine the most successful strategy particularly for recruiting from diverse ethnic groups.

### Eligibility screening

The eligibility of respondents will be assessed in a two-step sequential screening process:

1. *Phone-based screening:* After gaining verbal consent a preliminary phone screen will check inclusion and exclusion criteria that can be assessed by phone (e.g. self-reported inability to walk across a room), including a second check on medical exclusion criteria (e.g. recent heart surgery). Ability to attend intervention sessions will also be checked. Potential peer-volunteers will be contacted by telephone to check inclusion and exclusion criteria (age >  = 55 years, not in full-time employment unless working flexibly), and to collect their demographic data (Additional file 5).
2. *Face-to-face screening sessions:* Potentially eligible participants and peer-volunteers will then be invited to a group-based assessment session. The SPPB gait speed test will be conducted first, and those who fail to complete the 4-m walk or do not meet the other SPPB inclusion criteria will be thanked for their time and signposted to other suitable activities/services.

### Consent

Older adults who are willing to take part in ACE will be asked to provide written informed consent to the local research team prior to commencement of the face-to-face screening sessions (Additional file 6).

### Randomisation/allocation

Eligible participants will be randomised to one of the two arms in a 1:1 ratio stratified by site (West Midlands, Greater Manchester, South Wales and Bristol), using a centralised web-based system run by the Cardiff University Centre for Trials Research (CTR).

To perform randomisation an authorised member of the research team will access the randomisation website using unique username and password log-in details. The website will require entry of patient’s initials, date of birth and stratification variable. The randomisation website will also generate a unique study ID number for the participants when they are randomised. In the relatively unlikely event that two people from the same household present for screening, to avoid the potential for contamination (if they were allocated to different groups), only the first would be included in the study. This, and the reasoning behind it, will be carefully explained during the telephone screening process.

### Implementing the allocation sequence

The local site Research Associate will telephone participants to inform them of their allocation and will send a confirmation letter. The local site Research Associate will therefore not be blinded to allocation. Main outcome assessors at 6 and 18 months will be blinded.

To maximise retention, we will offer a voucher-based incentive for completion of assessments at 6 and 18 months. The control group will also be invited to two social events with a presentation on healthy ageing to encourage continuing engagement in the trial. Newsletters will also be distributed to all participants at the end of each project year.

### Intervention

Participants in the intervention arm will receive a 6-month active ageing programme, using peer volunteers to deliver individually tailored, and person-centred support to help inactive, less mobile older people to ‘get out and about’ to engage with their local community, with the aim of increasing physical activity. This is intended to lead to improvements in their mobility (specifically lower limb physical functioning) [14], as well as improvements in their physical and mental well-being and social connectedness.

1. Peer volunteers will meet participants twice in one-to-one meetings supporting them to identify local activities of interest and address barriers to participation (motivation stage: first 2 weeks). The particular relevance/benefits of activities that might improve lower limb physical function (i.e. those including a significant strength and balance component) will be discussed.
2. The volunteer-participant pair will attend at least three local initiatives chosen by the participant (action stage: month 1–3). We are collaborating with exercise providers for older adults in all three sites to ensure participants have opportunities to attend activities that specifically target lower limb physical function.
3. Telephone support to continue attending local activities. At least two further joint visits will be scheduled as support “tails off” (maintenance stage: months 3–6).

#### Control arm

After completion of baseline assessments, participants allocated to the control group will be invited to two, 60–90-min group sessions over the 18 months of the study. The sessions will be scheduled close to the 6- and 18-month assessment points and will consist of presentations and discussions on various aspects of healthy ageing (excluding physical activity).

### Underpinning theory

ACE draws on the Process Model of Lifestyle Behaviour Change (PMLBC) and Self Determination Theory [16, 17]. These two overlapping and mutually compatible theoretical frameworks provide the main principles and processes for supporting behaviour change in the ACE intervention. The ACE logic model illustrates how the intervention is theorised to lead to changes in short and longer-term outcomes, and how contextual (including system-level) processes might influence its operation (Additional file 3). These factors are consistent with qualitative feedback obtained from volunteers in the ACE feasibility study [14].

The ACE volunteer training is designed to teach volunteers how to use person-centred communication skills to build and sustain the motivation and engagement of the volunteers, through self-efficacy, positive feedback and fulfilment of the volunteer’s needs and expectations. It also includes teaching volunteers to guide participants along an individually tailored “road map” for behaviour change, based on three key stages: getting motivated; making a plan; and supporting behaviour maintenance.

Peer volunteers will attend a one-day training course delivered by ACE researchers. The course was developed and tested in the ACE feasibility study [14], and further refined in the ACE internal pilot in consultation with the Royal Voluntary Service. It will include (i) skills for developing and reinforcing motivation (person-centred counselling for supporting fundamental (SDT-related needs); (ii) how to identify local activity options and develop individually tailored plans based on participants’ needs /preferences; (iii) the need to build lower limb function (strength and balance) and types of exercise /activity associated with this; (iv) solution-focused methods for avoiding /overcoming barriers; and (v) maintenance support techniques. The training programme emphasises that the ACE volunteer’s role is to support the individual in becoming autonomous and responsible for making decisions about physical activity and social engagement within their local communities.

ACE volunteers will be managed and supported by local volunteer management partners such as RVS and AGE UK Bristol. NHS/government COVID-19 guidelines will be adhered to throughout all phases of the ACE intervention.

#### Steps to ensure intervention fidelity

We will enact strategies suggested by the NIH Behaviour Change Consortium to support intervention fidelity [18]. We will ensure “design fidelity” and “training fidelity” by building our intervention and training course around a clear logic model (Additional file 3). To support “delivery fidelity” we will encourage regular meetings between volunteers in order to (i) share their experiences and best practice, (ii) refresh principles covered at training and (iii) address any queries they have. To support “receipt” and “enactment” (understanding and use of behaviour change strategies by participants), we will encourage individual tailoring of intervention delivery by the facilitators, and reviewing of progress (including use of self-monitoring, sustainability-focused action-planning and problem-solving).

### Measures

A full list of measures and time-points are presented in Table 2. A copy of the Participant Case Report Form is included in Additional file 7. All questionnaires will be checked for completeness prior to participants leaving the assessment. Any missed questions will be brought to the attention of the participant for completion.

**Table 2:**
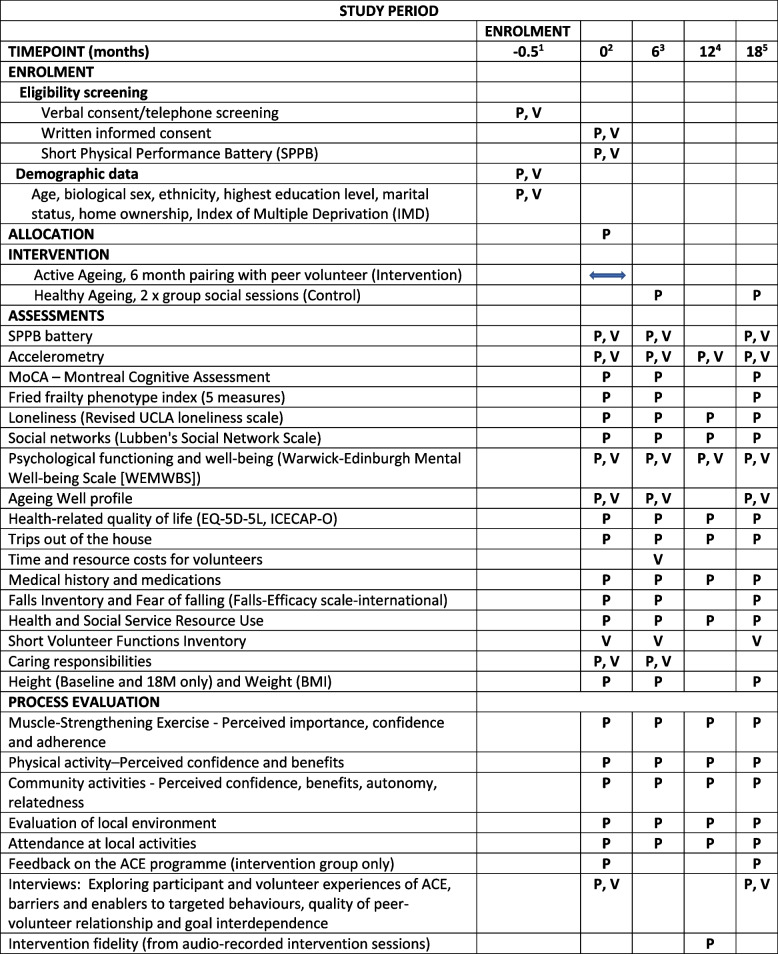
ACE screening and assessments for participants (P) and peer volunteers (V)

^1^Telephone screening, ^2^Face-to-face screening and baseline assessment, ^3^6-month follow-up, ^4^12-month follow-up, ^5^18-month follow-up

### Sample size calculation

The ACE trial will recruit a total of 515 participants across four study sites.

Effect Size: The primary aim is to assess the long-term (18-month) effect of a peer volunteering/physical activity intervention on changes in SPPB scores. A difference of 0.5 points in the SPPB score has been defined as a minimum clinically meaningful change [8, 19].

Using baseline data from 777 adults with the same inclusion criteria as we propose for this study (from our REACT trial [20, 21]), the standard deviation for SPPB scores in this population is 1.56. This is identical to the standard deviation observed for SPPB scores in the US-based LIFE trial (SD = 1.6, *N* = 1635). To detect a difference of 0.5 points in SPPB with 90% power and 5% significance, 206 participants are required per arm. Assuming a 20% loss to follow-up at 18 months [based on a 19% loss to follow-up at 24 months in our REACT study], the total sample size required is 515.

We will recruit a minimum of 130 volunteers (aged 55 + years) to support two intervention participants each. Since the cluster sizes in the intervention arm are minimal (less than two participants to each volunteer on average) we will not inflate the sample size required for clustering at the level of volunteer. However, the analytic strategy will present results from a partial cluster model to assess the small amount of possible clustering in the intervention arm only.

### Statistical analysis

Primary outcome analysis will be undertaken blinded to group allocation and will be on an intention-to-treat (ITT) basis with due emphasis placed on confidence intervals. Using appropriate descriptive statistics we will assess any imbalance between the trial arms at baseline and describe the characteristics of participants. The primary analytic strategy will employ a linear mixed model including baseline, 6- and 18-month SPPB outcome time points as well as the randomisation stratification variable (site). Treatment and time variables together with a categorical treatment by time interaction will be fitted to explore treatment effects over time. All results will be presented with 95% confidence intervals and *p*-values. A partial cluster regression model to account for the possible clustering effects of participants within volunteers will also be fitted to the primary outcome within the mixed model framework. Intra-cluster coefficients will be estimated and reported if non-negligible.

Depending on the extent of missing primary outcome data, the primary analysis will be repeated using the complete data set generated using multiple imputations. Sensitivity analyses will be conducted to investigate the potential effects of non-random missing data and of any co-interventions on the results and conclusions.

Secondary outcome analysis will be undertaken using the same general approach as for the primary analysis, using the baseline, 6-, 12- and 18-month follow-up data. This will include linear or generalised linear mixed regression models for continuous or binary outcomes as appropriate.

#### Sub-group analyses

Subgroup analyses are not powered for in this trial analysis but will be included and interpreted as exploratory only. Key pre-specified moderators of interest are area deprivation and ethnicity. If numbers allow, a group moderator interaction term will be added to the primary analysis model to investigate included moderator effects on treatment effectiveness. Other moderators to be considered for exploratory analysis include age group, sex, fall risk category and level of education.

#### Adjusted analysis

While we are not expecting a significant imbalance between groups, variables such as age and biological sex may be considered for inclusion in the primary model.

### Economic evaluation

The economic evaluation will estimate the incremental cost and incremental benefits of the ACE intervention compared to control, from a societal perspective. A within-trial analysis will estimate the cost-effectiveness at 18 months. A longer-term analysis will estimate the cost-effectiveness using a decision analytic model.

The within-trial analysis will adopt a micro-costing approach to estimate the costs related to the intervention. Study logbooks, questionnaires and volunteers’ time and travel diaries will collect resource use information at baseline, 6, 12 and 18 months for volunteer and participant time and travel, and health and social care use. Associated training costs will also be measured and reported separately. The resource use will be combined with unit cost data obtained from published sources such as the NHS reference costs [22], the British National Formulary [23] and the Prescription Cost Analysis [24] for England. Volunteer and participant time will be converted to costs using the value of leisure time. The primary outcome measure for the economic analysis will be quality-adjusted life year (QALYs) derived from utility scores, obtained from the EQ5D-5L instrument [25], and measured using the area-under-the-curve approach. Secondary economic outcomes will be Years of Full Capability (YFC), obtained from the ICECAP-O measure [26]. Both QALYs and YFC will be measured for the participants and volunteers. Any differences in baseline utility and capability index scores will be adjusted for in the analysis.

The economic evaluation will take the form of a cost-utility analysis (CUA) where the result will be expressed as a ‘cost per QALY’. In addition, a ‘cost per capability’ (CCapA) achieved will be estimated by combining the cost with improvements in capability. The analysis will report results of the CUA and CCapA from a health and social care perspective, including costs related to health and social care and QALYs/Capabilities accruing to the participants. Then, the analysis will be broadened to take a societal perspective including societal costs and outcomes for both participants and volunteers. Missing data will be explored and imputed using the most appropriate imputation technique that will depend on the nature of the missingness. Sensitivity analysis will be conducted to explore any uncertainties surrounding the key parameters using bootstrapping methods to estimate confidence intervals around the incremental cost-effectiveness. Cost-effectiveness acceptability curves will be generated to estimate the probability of the ACE intervention being cost-effective at different threshold willingness to pay values for a QALY. CHEERS guidelines will be followed for reporting the economic evaluations [27].

If the ACE intervention shows superiority in terms of improvements in mobility, we will conduct evidence synthesis and decision-analytic modelling to assess the lifetime cost-effectiveness of the intervention versus control, including consequences in terms of health and social care costs. Methods will follow best practice guidelines for decision-analytic modelling in health technology assessment [28].

### Process evaluation

The ACE process evaluation plan follows the principles of the UK Medical Research Council guidance on process evaluation [29] and aims to:

1. *Evaluate the quality and quantity of intervention delivery to inform conclusions about intervention effectiveness*
  To assess the quality of delivery of the ACE intervention we will:
  1.1. Audio-record participant-volunteer consultation meetings for a purposive sample of up to 30 pairs. Participants will be selected to achieve diversity in terms of age, biological sex and prior physical activity promotion experience. We will apply a fidelity checklist to code the data. This will include items to assess the quality and quantity of delivery of intervention processes that are part of the theory and logic model underpinning the ACE intervention. Scoring will be based on the Dreyfus scoring system for assessing clinical consultation skills [30]. Scoring will be completed by two coders independently. Encrypted digital audio recording devices will be used to record the consultations. Volunteer-participant contact time (intervention dose) will also be recorded and related to outcomes.
  1.2. Conduct semi-structured individual interviews with up to 30 volunteers and volunteer managers (all volunteer managers at each site) to identify variations in intervention delivery, provide feedback on the ACE training and implementation challenges. We will purposively sample volunteers to achieve diversity in age, biological sex, ethnicity and area deprivation and include volunteers who drop out of the programme.
2. *Investigate the proposed mechanisms of change, outlined in the ACE logic model and seek alternative explanations if this model is not supported*
  2.1. Administer brief questionnaires to participants and peer-volunteers at the baseline, 6-, 12- and 18-month data collection points (see measurement schedule, Table 1). The questionnaire data will be used to test hypotheses derived from the ACE logic model (Additional file 4: Appendix 4). This will include checking for between-group changes in the process variables listed in the model and mediation and moderation of the effects of the ACE intervention on the primary outcome, by changes in the process variables.
  2.2. Audio-record semi-structured interviews at 6 and 18 months with up to 30 participants, purposively selected for diversity in age, biological sex, ethnicity and functional status to identify whether the theorised mechanisms outlined in the logic model are evident in participant experiences and to identify ways in which the logic model could be refined. The data and data analysis will be integrated with that from volunteers/volunteer managers to further refine the logic model.

Analysis: The audio recordings will be transcribed verbatim and organised using the computer software NVIVO. All qualitative data will be analysed using thematic analysis, using the ACE logic model as an initial framework for coding.

3. *Understand the role of context and systems to inform whether and how the findings can be generalised.*
  We will identify systems-level and contextual barriers and facilitators of engagement with the intervention. This will inform future implementation of the intervention.
  3.1. Prior to the delivery of the ACE intervention, the researchers, service users and a wide range of stakeholders will co-produce a systems map [14, 31] to generate a comprehensive model, outlining the interplay between individual, systemic and contextual factors influencing physical activity and social engagement of the target population — and how this complex community-based public health intervention interacts with/affects these systems over time.
    This will be achieved via a series of audio-recorded half-day workshops held in each implementation site with (a) older people representing the target group who are not participating in ACE, and diverse in age, biological sex, functional ability, deprivation, ethnicity); (b) people with experience of volunteering to support older people; and (c) key active ageing agencies and organisational stakeholders (e.g. local authority service managers, VCSE organisations, public health, social care workers, activity session leaders, GPs and community nurses). The initial systems map will be used to (1) to refine the ACE intervention-delivery and recruitment strategies and (2) identify opportunities for enhanced joined action to support ACE delivery in each community.
  3.2. At the first stakeholder workshop, participants will be given a brief survey of relationships. This will ask them to identify the names and organisations of people who they consider important in their work in promoting physical activity with older people. This will be turned into a Social Network Analysis diagram using KUMU software [32]. This shows the extent and strength of relationships across the network and identifies key agencies or individuals who are central to the effectiveness of the network.

Both the methods above will be repeated post-intervention (with the same group of participants (the ‘map makers’) as well as with a group of up to 15 ACE participants and peer volunteers. This will identify changes and mechanisms of change in local systems and any strengthening of social networks. We will particularly seek to identify changes relating to the introduction of the ACE intervention into the community (if any). Changes in social networks will be shown through changes in key Social Network Analysis measures including degree; closeness; and betweenness measures [33].

We will seek to enhance the trustworthiness and depth of the analysis by inviting participants’ feedback on summaries of the analysed data. Our PPI group will also be involved in the interpretation of the data through workshops to discuss transcripts and the researchers’ interpretations of the data.

### Oversight and monitoring

The ACE trial will be overseen and monitored by a Trial Steering Committee (TSC), Data Monitoring and Ethics Committee (DMEC) and a Trial Management Group (TMG).

#### Trial Steering Committee (TSC)

The TSC will consist of an independent Chair with expertise in ageing and public health; the CI; at least one Advisory Group representative, an independent medical advisor, an independent statistician, an expert in health economics, a research expert from UKActive, plus two independent academics experienced in the design, delivery and evaluation of health promoting interventions in primary care and in the community. PIs at each site and health economist may be called on to attend as needed. The TSC will meet every 6–9 months from the start of the trial, providing overall supervision of the trial, monitoring trial progress and advising on scientific credibility. The TSC will consider and act, as appropriate, upon the recommendations of the Data Monitoring and Ethics Committee (DMEC) and will have the responsibility for deciding whether the trial needs to be stopped on grounds of safety or efficacy. The TSC will be blinded to all information regarding treatment assignments until the database is locked for final analysis or if the DMEC recommends that results need to be reviewed.

#### Data Monitoring and Ethics Committee (DMEC)

A fully independent DMEC will report to the TSC. This will comprise of an independent chair plus two senior academics including a statistician. The CI, PIs and Senior trial statistician may be invited to attend to provide specific input by the DMEC Chair with the CI and statistician usually expected to attend the ‘open session’ section of the meetings. The DMEC will be responsible for the interests of the participants and its main role will be to make recommendations to the TSC as to whether the trial needs to be stopped for any ethical or safety reason (based on review of accumulating safety data). The DMEC will undertake safety data reviews every 12 months after recruitment begins, unless otherwise deemed necessary. This will include data on any adverse advents reported during the trial.

The DMEC will be responsible for identifying any need for unblinding. The DMEC will also periodically review unblinded overall safety data to determine patterns and trends of events, or to identify safety issues, which would not be apparent on an individual case basis.

Analysed data will be blinded, unless the DMEC identifies a specific need for unblinding. The DMEC will meet shortly before the TSC and will provide a report for review during the TSC meeting.

#### Trial Management Group

The TMG will consist of the CI, all co-applicants, the trial manager, two people from our Advisory Group and the researchers at each trial centre. It will meet 4 times per year to ensure accurate implementation of the study protocol and the successful conduct and completion of the study.

Following the INVOLVE guidelines (involve.org.uk), we will have Advisory Group representatives on our Trial Management Group and Trial Steering Committee.

### Safety

The Trial Steering Committee and the Data Monitoring and Ethics Committee will oversee all patient safety issues, which the ACE independent medical advisor, will review in detail. All serious adverse events (SAE) will be examined by an independent medical advisor to see if they are related to the study intervention or measurement procedures. The ethics committee, the sponsor and the Trial Steering Committee or DMEC will be notified promptly (within 24 h) of all related serious adverse events.

Any adverse event or adverse reaction will be regarded as serious if it results in death; is life-threatening; requires non-elective hospitalisation, prolongation of existing hospitalisation or elective hospitalisation that may be related to taking part in the study; results in persistent or significant disability or incapacity. Only SAEs related to the study will be reported. The CI will maintain a register of all reported serious adverse events.

### Auditing trial contact

Participating sites will be required to permit a representative of the TSC or representative of the sponsor, to undertake study-related monitoring to ensure compliance with the approved study protocol and applicable SOPs, providing direct access to source data and documents as requested.

All study procedures will be conducted in compliance with the protocol and according to the principles of the International Conference on Harmonisation Good Clinical Practice (ICH GCP). Procedures specifically conducted by the CTR team (e.g. randomisation) will be conducted in compliance with CTR standard operating procedures (SOPs).

### Confidentiality

Local contact databases containing participant and volunteer names and addresses will be created at each site for the purpose of managing appointments, questionnaires, intervention delivery and process evaluation interviews. These will be stored at each site in a SQL server database, housed on a restricted access, secure server. Investigators will ensure that the participants’ anonymity is maintained on all paper documents through the use of Participant IDs and the storing of anonymised and identifiable study data separately. Identifiable study data will be stored in locked filing cabinets within a locked office.

Data collected via the ACE screening form and the CRF will be entered, by members of the research team, onto one central data entry website developed by Cardiff Centre for Trials Research and will be encrypted using SSL.

### Ancillary and post-trial care and compensation to those who suffer harm

The University of Birmingham has arranged Public Liability insurance to cover the legal liability of the University as Research Sponsor in the eventuality of harm to a research participant arising from the management of the research by the University.

### Dissemination plan

Dissemination will commence from project initiation with the creation of a project website and will be planned according to NIHR guidance.

1. Our ACE/Active Ageing website (https://www.activeageingresearch.org/about-ace) will publish ACE news and progress.
2. Showcase events will be delivered at all sites after completion of ACE to present the findings and celebrate successful lifestyle change stories as told by peer volunteers and participants themselves
3. ACE Infographics will be developed to provide an appealing and accessible, graphical description of the study
4. A Pop-up ACE stand will be created to increase visibility at events, conferences, exhibitions
5. At least 5 papers will be submitted for publication in peer-reviewed journals, including 3 open access journals
6. Presentations will be delivered at least two academic conferences and at third sector organisation annual meetings and events organised by local partners
7. A brief promotional piece to be mailed to all UK Directors of Public Health and other key decision-makers in voluntary sector organisations promoting the use of the delivery toolkit
8. Newsletters will be distributed to participants at the end of each project year and to academic and non-academic partners
9. Social media (including Universities’ Twitter accounts and Facebook pages) and local media (newspapers, magazines) will be used to publish news briefings

## Discussion

Breaking the spiral of age-related decline that is characterised by loss of physical and cognitive function, reduced capacity to independently manage daily tasks, and reductions in social interaction is fundamental to healthy ageing. It also has the potential to substantially reduce reliance on health and social care services. This is particularly true for those who are at risk of mobility-related disability resulting from low levels of physical activity as they settle into changed routines after their primary working years.

Prospective cohort studies and trial data demonstrate that both moderate and lighter-intensity physical activity are associated with a lower risk of mobility disability [34]. An active older person has a 36% lower risk of developing functional limitations and a 38% lower risk of hip fracture [35]. The wider health and well-being benefits associated with physical activity in older age are well documented [36]. However, in the UK, levels of activity decrease with age, with 47.9% of people aged 65 years and older being classified as inactive [37].

Recent policy documents, including the NHS Long Term Plan, call for new service models to proactively support older people living with frailty in the community [38, 39]. A consensus statement led by Public Health England identifies five key commitments towards healthy ageing: prioritising prevention initiatives; removing barriers and creating more opportunities for older adults to contribute to society; adopting a range of community-centred approaches that support and encourage community participation; narrowing inequalities and challenging ageism [40]. The ACE study aligns with these commitments, is relevant to policies targeting living independently in the community and has strategic importance for social and health care policy across the UK. The voluntary sector is an untapped resource, ideally placed to deliver low-cost and effective interventions and to increase access to disadvantaged populations [41, 42]. However, there have been few high-quality trials evaluating community approaches that mobilise peer volunteers to promote active ageing and none that specifically target people at risk of mobility disability [43].

ACE is the largest, pragmatic (defined as measuring effectiveness in real-life settings) [44], community-based trial in the UK to target this high-risk segment of the older population by mobilising community resources (peer volunteers). ACE is also the first study of its kind to conduct a full economic and comprehensive process evaluation alongside the RCT. If effective and cost-effective, the ACE intervention has strong potential to be implemented widely in the UK and elsewhere.

## Trial status

Enrolment into the study started in March 2022. Recruitment is expected to be completed by 31 December 2023 and final follow-up assessments by 31 May 2025. Protocol Version 5 dated 22 July 2022.

### Supplementary Information

**Additional file 1.** Completed SPIRIT (2013) checklist detailing on which page of the protocol manuscript each of the relevant recommended items is addressed.

**Additional file 2.** Description of the process by which the decision to progress the ACE study from pilot to main trial will be made.

**Additional file 3.** ACE Logic Model.

**Additional file 4.** ACE original ethical approval dated 11^th^ August 2021.

**Additional file 5.** ACE Telephone Screening Form used to collect demographic data and conduct initial eligibility assessment with potential participants.

**Additional file 6.** ACE Participant Consent Form.

**Additional file 7.** ACE Participant Case Report Form including measures and items used to collect outcome data as described in Table 2.

## Data Availability

The NIHR’s Policy on Open Access will be adhered to and data supporting published findings will be made accessible. Direct access will be granted to authorised representatives from the Sponsor, host institutions and the regulatory authorities to permit trial-related monitoring, audits and inspections. Access to the website will be overseen by the CI and Trial Manager. Subject to data protection provision (data to be anonymised), the Secretary of State for Health has the right to have access and use data collected and used for the purpose of the project.

## Abbreviations

AE: Adverse event
CI: Chief Investigator
CEA: Cost-effective analysis
CRN: Clinical Research Network
CRF: Case Report Form
CTC: Clinical Trials Centre
DMEC: Data Monitoring and Ethics Committee
GCP: Good Clinical Practice
ICF: Informed Consent Form
ISRCTN: International Standard Randomised Controlled Trials Number
ITT: Intention-to-treat
MVPA: Moderate to vigorous physical activity
PI: Principal Investigator
PIC: Participant Identification Centre
PIS: Participant Information Sheet
QALY: Quality-adjusted life year
RCT: Randomised control trial
REC: Research Ethics Committee
SAE: Serious adverse event
SOP: Standard operating procedure
SPIRIT: Standard Protocol Items: Recommendations for Interventional Trials guidelines
SPPB: Short Physical Performance Battery
TMG: Trial Management Group
TSC: Trial Steering Committee
VSCE: Voluntary, Community or Social Enterprise
