# Supplementary material for: A peer-volunteer led active ageing programme to prevent decline in physical function in older people at risk of mobility disability (Active, Connected, Engaged [ACE]): study protocol for a randomised controlled trial
Source: Trials. 2023 Nov 29;24:772. doi: 10.1186/s13063-023-07758-3 (PMC10687817; doi:10.1186/s13063-023-07758-3)
Supplement: Supplementary file 2 — Additional file 2. Description of the process by which the decision to progress the ACE study from pilot to main trial will be made. [file 13063_2023_7758_MOESM2_ESM.pdf]

Additional file 2 Progressing from the Internal Pilot to the Main Trial

The Trial Steering Committee, with advice from the Data Monitoring and Ethics Committee, will assess the feasibility of the trial during the internal pilot phase, taking into account findings on the acceptability of trial procedures, intervention adherence and recruitment and retention rates. Based on our recruitment rates in previous UK-based physical activity interventions with similar target populations (Project ACE, Better Ageing) and with equivalent of 1FTE research assistants at each of three sites, in the pilot study we anticipate a recruitment rate of 15 participants/month/site, (180 participants will be recruited in total over 4 months). If the recruitment rate is less than predicted in a given month, we will take actions to increase it (increasing the number of people approached and/or increasing the geographical area, adapting recruitment procedures). After 6 months, recruitment data will be reviewed by the TSC and any required changes in the recruitment strategy and/or introduction of new recruitment avenues will be discussed and agreed. Retention rates (proportion of people providing follow up data) will also be checked at 6 months. Receipt of strong negative feedback from the majority of either participants or intervention providers about the intervention or trial methods will be considered as a stopping criterion. The participants recruited in the pilot study will be included in the trial analysis.
