# Supplementary figures and images for: A peer-volunteer led active ageing programme to prevent decline in physical function in older people at risk of mobility disability (Active, Connected, Engaged [ACE]): study protocol for a randomised controlled trial
Source: Trials. 2023 Nov 29;24:772. doi: 10.1186/s13063-023-07758-3 (PMC10687817; doi:10.1186/s13063-023-07758-3)

## Additional File 3 ACE Logic Model

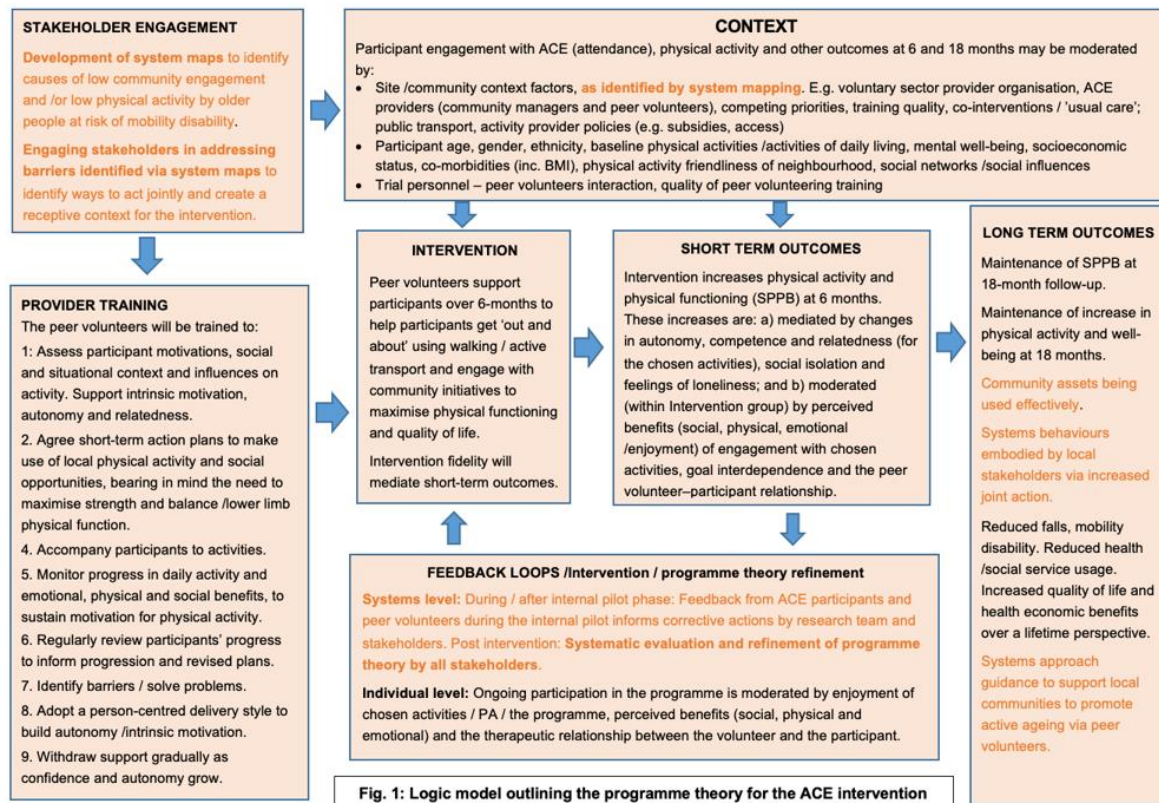

Supplement: Supplementary file 3 — Additional file 3. ACE Logic Model. [file 13063_2023_7758_MOESM3_ESM.pdf]
