# Supplementary material for: A peer-volunteer led active ageing programme to prevent decline in physical function in older people at risk of mobility disability (Active, Connected, Engaged [ACE]): study protocol for a randomised controlled trial
Source: Trials. 2023 Nov 29;24:772. doi: 10.1186/s13063-023-07758-3 (PMC10687817; doi:10.1186/s13063-023-07758-3)
Supplement: Supplementary file 5 — Additional file 5. ACE Telephone Screening Form used to collect demographic data and conduct initial eligibility assessment with potential participants. [file 13063_2023_7758_MOESM5_ESM.pdf]

ACE: Participant Screening V3

Date of Screening:	Researcher:		
Participant ID:	Date of birth		
Verbal consent for telephone screening given		Yes	No
Biological sex		Male	Female

GP Details

GP Surname: GP 1st Name:

Practice name:

Address:

Telephone number:

If the participant doesn't know all GP details (i.e. telephone number) complete these using an internet search after the telephone screening call. Some people will not see a specific GP in which case just record the GP practice details.

1. How did you hear about the ACE study?

**2. Ethnicity**<br>i. White – British<br>ii. White – Irish<br>iii. Any other White background<br>iv. Mixed – White and Black Caribbean<br>v. Mixed – White and Black African<br>vi. Mixed – White and Asian<br>vii. Any other Mixed background<br>viii. Asian or Asian British – Indian<br>ix. Asian or Asian British – Pakistani<br>x. Asian or Asian British – Bangladeshi<br>xi. Any other Asian background<br>xii. Black or Black British – Caribbean<br>xiii. Black or Black British – African<br>xiv. Any other Black background<br>xv. Chinese<br>xvi. Any other ethnic group (please state) _______________	(record number here)

**3. What was your <u>highest</u> education level completed?**<br>1. Primary school<br>2. Secondary school<br>3. Some college or vocational training<br>4. Completed college<br>5. Completed university<br>6. Completed post-graduate degree, or higher			(record number here)	
**5. What type of residence do you live in?**<br>1. Bungalow<br>2. Terrace house<br>3. Semi-detached house<br>4. Flat<br>5. Detached house<br>6. Assisted community home, including sheltered housing<br>**7.** Other (please specify			(record number here)	
**6. Do you rent or do you own your own home?**<br>1. Own/buying<br>2. Rent<br>*3.* Neither (living with relatives etc.)				
**7. What is your marital status?**<br>1. Married<br>2. Widowed<br>3. Divorced/separated<br>4. Single and never married<br>5. Living with partner<br>6. Other (please specify)<br>**Including you, how many people live in your household**<br>1. One<br>2. Two<br>3. Three or more				
"Now I'm going to run through a series of questions and when we get to the end I'll explain whether you could be eligible to take part in ACE and if you are we'll go onto a few more questions."			Yes	No
8. Are you in full time work?			Yes	No
9. A) Do you use a wheelchair?			Yes	No
B) Do you use a Zimmer frame?			Yes	No
10. How would you find walking across a room? (With a walking stick is ok if needs a zimmer tick Unable)	Easy	A little difficult	Very difficult	Unable
11. How easy would you find getting out of a low chair?	Easy	A little difficult	Very difficult	Unable
a) If response is Easy, ask: Would you normally use your hands to help you get up from the chair?	Yes/No			
12. How easy would you find walking up a flight of stairs with no handrail or wall to lean on?	Easy	A little difficult	Very difficult	Unable
13. How easy do you find walking on an uneven pavement without losing your balance?	Easy	A little difficult	Very difficult	Unable

**Is the participant Unable to walk across a room (Q10)?**<br>**If 'yes', explain that:** *"Unfortunately, we can't include you in the ACE programme at the moment as our participants need to be able to get out and about to local activities. Thank you very much for your time.*	Yes	No
**Does participant find <u>all four</u> of these easy and answered No to 12a?**<br>**If 'yes', explain that:** *"Unfortunately, we can't include you in the ACE programme at the moment (use the explanation in the Screening script, including asking if they would consider being a peer volunteer). Thank you very much for your time.*	Yes	No
14. Has a doctor ever advised you not to exercise?<br>**<u>If yes</u>**, Why was that? (See researcher notes for advice depending on answer)	Yes	No
15. Are you planning to move out of the area within the next 18 months?	Yes	No
16. Do you live in a residential care home or a nursing home?	Yes	No
17. Have you been diagnosed by your GP with any of the following medical conditions:		
a) Arthritis (either osteo or rheumatoid) that is so severe that it would prevent you from walking about 100 yards/metres, that's about the length of a football pitch	Yes	No
b) Parkinson's disease	Yes	No
c) Dementia	Yes	No
d) Lung disease that requires using oral steroids (tablets) or supplemental oxygen? (Does not include using an inhaler)	Yes	No
e) Severe kidney disease that requires dialysis	Yes	No
f) Chest pain when walking one or two hundred yards or up a flight of stairs	Yes	No
g) Do you have an implanted cardiac defibrillator?	Yes	No
h) Have you ever had a cardiac arrest which required resuscitation?	Yes	No
i) Within the last six months have you had major heart surgery, including valve replacement or bypass surgery?	Yes	No
j) Do you have any other heart condition? **<u>If yes</u>** please specify:<br>____	Yes	No

k) Are you currently receiving radiation treatment and/or chemotherapy for cancer *Interviewer Note: Tamoxifen for breast cancer or hormonal therapy for any cancers is not chemotherapy*	Yes	No
l) Are you awaiting knee or hip surgery?	Yes	No
m) Within the last six months have you had spinal surgery?	Yes	No
n) Have you been diagnosed with a terminal illness	Yes	No
18. Are you planning to go into hospital any time in the next year for anything other than hip or knee surgery?	Yes	No
**Do any of the participant's answers for Qu 14-18 fall into any of the shaded boxes?**	Yes ▾	No

If 'yes', explain that:

"Unfortunately, we can't include you in the ACE programme at the moment (use the explanations in the Screening script), but thank you very much for your time. We will send you some information about Healthy Ageing that you might find useful.

If being unable to walk across a room without assistance is due to a temporary condition record the temporary condition and treat as a temporary exclusion.

Condition: ____

Date to call back: ____

If no answers fall into the shaded boxes explain that:

"You could be eligible to take part in ACE, but I will just ask you a few more questions"

**19. Do you drive?**	Yes	No
**If 'yes':** Do you have access to a car that you can use regularly?	Yes	No
**If 'no':** Do do you have access to other forms of transport?	Yes	No

**20. Confidence in ability to engage with the six-month ACE programme:**<br>*"None of us can predict exactly what will happen over the next year, holidays, illnesses and minor operations may crop up unexpectedly, but in general if you were allocated to the ACE programme, how confident do you feel that you would usually be able to meet with an ACE volunteer regularly (once a week at first) and regularly attend some local activities, activities that you choose, for the next six months".*

Quite confident	Not sure	Not at all confident

If not at all confident ask and record the reason(s) why:

____

If participant is not confident because of transport problems: *"If you are allocated to the ACE programme, we will match you with a volunteer who lives as close to you as possible. Would living close with the volunteer make you more confident about taking part?" (Indicate below if yes)*

So are you happy you would be able to meet with a peer volunteer on an one-to- one basis and attend activities together during the 6 months of the ACE programme?

If still not confident, suggest that: *"Would it be better if we called you back in a few months to see if you are feeling more able to take part?"*

Agreed to be called back<br>Date: _________________	Need transport to be arranged	Preferred not to be called back

Temporary exclusions

"I will just continue to ask a few more questions…"

21. Within the last 6 months have you had a hip fracture?	Yes	No
22. Within the last 6 months have you had a hip or knee replacement?	Yes	No
23. Within the last 6 months have you had a heart attack (or "myocardial infarction") that required overnight hospitalisation?	Yes	No
24. Within the last 6 months have you had a stroke (does not include a transient ischemic attack (TIA) or mini-stroke)?<br>Did this result in any movement related impairment?<br>____	Yes	No
25. Are you currently receiving physical therapy on your legs or to help with movement in your legs?	Yes	No
26. Are you currently enrolled in another physical activity research or intervention study? **If yes:**<br>Study name: ________________________________<br>Study end date:______________________________	Yes	No

If participant answers fall into any of the shaded boxes, explain that: *"We can't include you in the ACE trial at the moment, but if you are happy for us to do so, we will contact you again in a few months to re-assess the situation."*

If 'yes': *"Could you suggest a good date to call you back?"* Date:_________________

**If 'no':** *"That is not a problem. Would you like us to post you an information pack which contains details of local activities and places where you will able to get health advice?"*	Yes	No
**If *none* of the responses fall into shaded boxes:** *"Just a few last questions…"*	Yes	No
27. Is there a health reason not mentioned why you would be concerned about starting to be involved in more activities, or being a bit more active?<br>**If 'yes'** ask for more details:<br>____	Yes	No
28. If you are allocated to take part in the ACE intervention it would be useful for us to know a bit about your interests and hobbies, either now or in the past, so that we can try and match you with a volunteer with similar interests.		
29. And now just a few questions about your technology use. This will help us communicate with you in the way that suits you.<br>**a)** Do you use a mobile phone?	Yes	No
b) Is your phone a smart phone? (one with a touchscreen and access to the internet)	Yes	No
c) Do you use a computer or a tablet?	Yes	No
d) Do you use email?<br>If yes ask for email address ___________________________________-_____	Yes	No
e) Do you use Facebook, Twitter or WhatsApp? (circle those used)	Yes	No
f) Do you use Zoom or any other video meeting product?	Yes	No
30. And finally Is anyone else in your household taking part in ACE?<br><br>If yes please could you let me have their name? ………………………………….<br>*Just to let you know that we can't include two people from one household in the ACE study. So if it is ok with you we will conduct this screening with you both and move on to face to face screening. If you both meet the criteria for taking part in the study we will include the person whose reply form we received first. Is that ok with you?*	Yes	No

"Thank you. That's all I have to ask you at the moment, is there anything you'd like to ask me?"............................ "From what you have told me so far you could be eligible to take part in ACE so what we would like to do now is invite you to attend a session at (venue) so that we can meet face to face and conduct a few more simple screening tests such as asking you to walk 4 metres and answer a few more questions."

1. Are you still happy to take part in ACE?	Yes	No

2. **If still 'No':** *'That is not a problem at all, if you change your mind, please do get back in touch with us vial telephone, post or email.*

Explain the <u>time</u> and <u>venue</u> details of the screening session and discuss any <u>transport requirements</u>, explain travel expenses will be paid.

"It's been nice talking to you. I'll send you an email or post you a letter to confirm the details of the face-to-face screening session. Which would you prefer?" ***POST / EMAIL*** *Also if you do have any friends or relations who are over 65 years old who you think might be also be interested in taking part in ACE please pass on our contact details.*

"The confirmation letter/email I send you will include a short form asking about any medications you take. If you wouldn't mind could you complete that at home and bring it with you to the assessment session – please don't bring any medications with you to the session, just the completed form OK, that's it for today. If there are any more questions you want to ask you can call me anytime. Thank you very much for your time."

Researcher:-

- ☐ Record date details of measurement session sent ______________________
- ☐ Finalise transport arrangements. Transport details ______________________
- ☐ File Screening form
- ☐ Enter details onto database Date _____________________
