# Supplementary material for: A peer-volunteer led active ageing programme to prevent decline in physical function in older people at risk of mobility disability (Active, Connected, Engaged [ACE]): study protocol for a randomised controlled trial
Source: Trials. 2023 Nov 29;24:772. doi: 10.1186/s13063-023-07758-3 (PMC10687817; doi:10.1186/s13063-023-07758-3)
Supplement: Supplementary file 6 — Additional file 6. ACE Participant Consent Form. [file 13063_2023_7758_MOESM6_ESM.pdf]

# ACE: Active, Connected, Engaged (known in Wales as ACTIF)

A multi-centre randomised controlled trial of a peer volunteer led active ageing programme to prevent decline in physical function in older people at risk of mobility disability

| | | | |
|---|---|---|---|
| Centre: | | Participant number: | |

Chief Investigator: Professor Afroditi Stathi, University of Birmingham

## Participant Consent Form

Please look at each of the statements and decide whether or not you agree. If you agree please sign your **initials** in the box, if you don't agree leave the box blank.

| | |
|---|---|
| I confirm that I have read and understood the contents of **The ACE Study: Active, Connected, Engaged** Participant Information Sheet dated ………. (Version….) and have had the opportunity to consider the information, ask questions and have received satisfactory answers. | |
| I understand that the information collected about me will be stored anonymously with a numeric code, which means I cannot be identified by anyone outside of the research team. The data will be destroyed after 15 years from study completion. | |
| I agree to my GP being informed of my participation in the study, and to my GP being contacted if the research team become concerned about my health or well-being. | |
| I consent to my contact details, and data collected during the screening process, being shared with (*the local ACE volunteer management partner, i.e. Royal Voluntary Service*) to help match me with a peer volunteer if I am randomly allocated to the intervention group. I agree to be contacted for the purpose of this study. | |
| I understand that relevant sections of my medical notes, and data collected during the study, where it is relevant to my taking part in this research, may be looked at by individuals from the Universities involved in the study (Birmingham, Manchester, Cardiff Metropolitan, Cardiff, Exeter and Bath), (*the local ACE volunteer management partner, i.e. Royal Voluntary Service*) and by regulatory authorities (for the purpose of monitoring and audit only). I give permission for these individuals to have access to my records. | |



NHS National Institute for Health Research

| | |
|---|---|
| I consent to my data being stored for use in future ethically approved research (Optional). | |
| I understand that taking part is voluntary and that I can change my mind, withdraw from **any part** of the study, at **any** time without giving any reason, without penalty and without my medical care or legal rights being affected. | |
| I understand that data collected about me and information I provide during discussions might be used in a written report and publications. My name will not be used and it will not be possible for anyone outside of this study to identify me. | |
| I agree to be approached to participate in a one-to-one or group discussion where we will discuss the ACE project. I understand that these discussions will be recorded and agree to the audio-recording of this discussion. If happy to participate, I will provide my verbal consent prior to the start of the discussion. | |
| I understand the assessments described within the information sheet and agree to take part in these assessments, including the monitoring of my physical activity and physical function tests. | |
| I agree to the audio-recording of a face-to-face meeting between me and an ACE peer volunteer. | |
| I consent to be contacted to discuss taking part in long-term follow up (up to 10 years) after the end of the study (Optional) | |
| I agree to take part in this study | |

| | | |
|---|---|---|
| ______________________ | ____________________ | ____________________ |
| Name of participant | Date | Participant's signature |
| ______________________ | ____________________ | ____________________ |
| Name of person taking consent | Date | Signature |

***Original copy for the study file, one copy for the participants' medical records, one copy for the participant***

Many thanks for your help
