# Supplementary material for: A peer-volunteer led active ageing programme to prevent decline in physical function in older people at risk of mobility disability (Active, Connected, Engaged [ACE]): study protocol for a randomised controlled trial
Source: Trials. 2023 Nov 29;24:772. doi: 10.1186/s13063-023-07758-3 (PMC10687817; doi:10.1186/s13063-023-07758-3)
Supplement: Supplementary file 7 — Additional file 7. ACE Participant Case Report Form including measures and items used to collect outcome data as described in Table 2. [file 13063_2023_7758_MOESM7_ESM.pdf]

Questionnaire (V3)

We need your help to make our study a success.
Remember that…

- **There are no right or wrong answers – please just be as honest as possible and go with your 'gut reaction'. Don't over-think your answers**
- **Everything you tell us will be kept strictly confidential (secret) and you're free to decline answering any questions you're not comfortable with**
-

For Researcher Use

Participant ID

Measures 1 ❒ Measures 2 ❒ Measures 3 ❒ Measures 4 ❒

Date: ______________________ Researcher initials: _________

Data entry researcher

ID CODE: ____________________ Initials: _______

SCREENING SECTION

Researcher: Do you think this participant is in the ***Intervention group*** ❐ or the ***Control group*** ❐?

A. SPPB Physical function tests (Baseline, 6M and 18M)

4m walking speed (☑) – Strike through following table if not used

ATTEMPT	Time (circle best of 2)	≤4.81s	4.82-6.20s	6.21-8.70s	8.71-59.99s	>60s/Unable	Aid used? (circle)	Code non-attempt/fail	SCORE
#1		❐ [4]	❐ [3]	❐ [2]	❐ [1]	❐ [0]	No / Stick / Other		
#2		❐ [4]	❐ [3]	❐ [2]	❐ [1]	❐ [0]	No / Stick / Other		

BASELINE ONLY (If participant does not complete the 4-metre walk they are not eligible to participate in ACE. Do not continue the tests. Explain the situation. Thank them for their time. Offer refreshments, ensure their transport back home is available.

Standing balance (☑)	Side-by-side	Semi-tandem	Full tandem stand	BALANCE SCORE (SUM)
**Time** (record *only if* <10s)				
**Code non-attempt /fail [0]**				
	**< 10 s** ❐ [0] END	< 10 s ❐ [0] END	< 3 s ❐ [0]	
	**≥ 10 s** ❐ [1]	≥ 10 s ❐ [1]	3-9.99 s ❐ [1]	
			≥ 10 s ❐ [2]	

Chair rise(s) (☑)

Completed single chair stand? (circle)	Time	≤11.19s	11.20–13.69s	13.70-16.69s	16.70-59.99s	>60s/Unable to complete 5 stands/used hands	Code non-attempt/fail	SCORE
Yes / No / Used hands [0]		❒ [4]	❒ [3]	❒ [2]	❒ [1]	❒ [0]		

3m walking speed (☑) – For home visits where 4M walk not possible. Strike through following table if not used

ATTEMPT	Time (circle best of 2)	≤3.61s	3.62-4.65s	4.66-6.52s	6.53-59.99s	>60s/ Unable	Aid used? (circle)	Code non-attempt/fail	SCORE
#1		❒ [4]	❒ [3]	❒ [2]	❒ [1]	❒ [0]	No /Stick /Other		
#2		❒ [4]	❒ [3]	❒ [2]	❒ [1]	❒ [0]	No /Stick /Other		

CODES for non-attempt or failure

1 Tried but unable
2 Participant could not hold position/stand/walk unassisted
3 Not attempted, researcher felt unsafe
4 Not attempted, participant felt unsafe
5 Participant unable to understand instructions
6 Other (specify in tables above)
7 Participant refused

PARTICIPANT'S FINAL SCORE	

BASELINE ONLY If participant scores lower than 4 or more than 9 they are not eligible to participate in ACE (a score of 4-9 is required to participate) do not continue the tests. Explain the situation. Thank them for their time. Offer refreshments. Spend some time explaining the information pack and ensuring their transport home is available.

C. Everyday activities (Baseline, 6M and 18M)

INSTRUCTIONS:

Please complete this questionnaire by either circling the correct response. Here is an example:

During the past 7 days, how often have you seen the sun?

[0.] NEVER [1.] SELDOM (1-2 DAYS) [2.] SOMETIMES (3-4 DAYS) [3.] OFTEN (5-7 DAYS)

Answer all items as accurately as possible. All information is strictly confidential.

LEISURE TIME ACTIVITY

1. Over the past 7 days, how often did you participate in sitting activities such as reading, watching TV or doing handicrafts?

[0.] NEVER → GO TO Q#2 [1.] SELDOM (1-2 DAYS) [2.] SOMETIMES (3-4 DAYS) [3.] OFTEN (5-7 DAYS)

1a. What were these activities?

____

____

1b. On average, how many hours per day did you engage in these sitting activities?

[1.] LESS THAN 1 HOUR [2.] 1 BUT LESS THAN 2 HOURS

[3.] 2-4 HOURS [4.] MORE THAN 4 HOURS

2. Over the past 7 days, how often did you walk outside your home or garden for any reason? For example, for fun or exercise, walking to work, walking the dog, etc.?

[0.] NEVER ▼ GO TO Q#3	[1.] SELDOM (1-2 DAYS) ▼	[2.] SOMETIMES (3-4 DAYS) ▼	[3.] OFTEN (5-7 DAYS) ▼

2a. On average, how many hours per day did you spend walking?

[1.] LESS THAN 1 HOUR [2.] 1 BUT LESS THAN 2 HOURS
[3.] 2-4 HOURS [4.] MORE THAN 4 HOURS

3. Over the past 7 days, how often did you engage in light sport or recreational activities such as bowling, golf with a trolley, fishing or other similar activities?

[0.] NEVER ▼ GO TO Q#4	[1.] SELDOM (1-2 DAYS) ▼	[2.] SOMETIMES (3-4 DAYS) ▼	[3.] OFTEN (5-7 DAYS) ▼

3a. What were these activities?

____

____

3b. On average, how many hours per day did you engage in these light sport or recreational activities?

[1.] LESS THAN 1 HOUR [2.] 1 BUT LESS THAN 2 HOURS

[3.] 2-4 HOURS [4.] MORE THAN 4 HOURS

4. Over the past 7 days, how often did you engage in moderate sport and recreational activities such as doubles tennis, ballroom dancing, golf without a trolley, low impact aerobics class (50 years +) or other similar activities?

[0.] NEVER ▼ GO TO Q#5	[1.] SELDOM (1-2 DAYS) ▼	[2.] SOMETIMES (3-4 DAYS) ▼	[3.] OFTEN (5-7 DAYS) ▼

4a. What were these activities?

____

____

4b. On average, how many hours per day did you engage in these moderate sport and recreational activities?

[1.] LESS THAN 1 HOUR [2.] 1 BUT LESS THAN 2 HOURS

[3.] 2-4 HOURS [4.] MORE THAN 4 HOURS

5. Over the past 7 days, how often did you engage in strenuous sport and recreational activities such as jogging, swimming, cycling, singles tennis, strenuous aerobic exercise class where you get very out of breath or other similar activities?

[0.] NEVER	[1.] SELDOM	[2.] SOMETIMES	[3.] OFTEN
GO TO Q#6	(1-2 DAYS)	(3-4 DAYS)	(5-7 DAYS)

5a. What were these activities?

____

____

5b. On average, how many hours per day did you engage in these strenuous sport and recreational activities?

[1.] LESS THAN 1 HOUR [2.] 1 BUT LESS THAN 2 HOURS

[3.] 2-4 HOURS [4.] MORE THAN 4 HOURS

6. Over the past 7 days, how often did you do any exercises specifically to increase muscle strength and endurance, such as lifting weights or push ups/press ups, using therabands (elasticated resistance bands) etc.?

[0.] NEVER	[1.] SELDOM	[2.] SOMETIMES	[3.] OFTEN
GO TO Q#7	(1-2 DAYS)	(3-4 DAYS)	(5-7 DAYS)

6a. What were these activities?

____

____

6b. On average, how many hours per day did you engage in exercises to increase muscle strength and endurance?

[1.] LESS THAN 1 HOUR [2.] 1 BUT LESS THAN 2 HOURS

[3.] 2-4 HOURS [4.] MORE THAN 4 HOURS

HOUSEHOLD ACTIVITY

Please circle your answer

7. During the past 7 days, have you done any light housework, such as dusting or washing dishes?
   [1.] NO [2.] YES

8. During the past 7 days, have you done any heavy housework or chores, such as vacuuming, scrubbing floors, washing windows, or carrying wood?
   [1.] NO [2.] YES

9. During the past 7 days, did you engage in any of the following activities? Please answer <u>YES</u> or <u>NO</u> for each item by circling your response.

		NO	YES
a.	Home repairs like painting, wallpapering, electrical work, etc.	1	2
b.	Lawn work or clearing leaves or snow etc.	1	2
c.	Outdoor gardening (i.e. digging or weeding)	1	2
d.	Caring for another person, such as children, dependent spouse, or another adult	1	2

WORK-RELATED ACTIVITY

Please circle your answer

10. During the past 7 days, did you work for pay or as a volunteer?

    [1.] NO   [2.] YES

If **NO** please go to **SECTION D**, if **YES** please give details below:

10a. How many hours per week did you work for pay or as a volunteer (in total)?<br>_____________________________ HOURS<br>10b. Which of the following categories best describes the amount of physical activity required on your job and/or volunteering work? Circle one number.<br>[1] Mainly sitting with slight arm movements. [**Examples:** office worker, watchmaker, seated assembly line worker, bus driver, etc.]<br>[2] Sitting or standing with some walking. [**Examples:** cashier, general office worker, light tool and machinery worker.]<br>[3] Walking, with some handling of materials generally weighing less than 40 pounds. [**Examples:** postman, waiter/waitress, construction worker, heavy tool and machinery worker.]<br>[4] Walking and heavy manual work often requiring handling of materials weighing over 50 pounds. [**Examples:** forestry work, stone mason, farm or general labourer].

D. EQ-5D-5L (Baseline, 6M, 12M and 18M)

Under each heading, please tick the ONE (✔) that best describes your health TODAY.

MOBILITY

I have no problems in walking about	☐
I have slight problems in walking about	☐
I have moderate problems in walking about	☐
I have severe problems in walking about	☐
I am unable to walk about	☐

SELF-CARE

I have no problems washing or dressing myself	☐
I have slight problems washing or dressing myself	☐
I have moderate problems washing or dressing myself	☐
I have severe problems washing or dressing myself	☐
I am unable to wash or dress myself	☐

USUAL ACTIVITIES *(e.g. work, study, housework, family or leisure activities)*

I have no problems doing my usual activities	☐
I have slight problems doing my usual activities	☐
I have moderate problems doing my usual activities	☐
I have severe problems doing my usual activities	☐
I am unable to do my usual activities	☐

PAIN / DISCOMFORT

I have no pain or discomfort	☐
I have slight pain or discomfort	☐
I have moderate pain or discomfort	☐
I have severe pain or discomfort	☐
I have extreme pain or discomfort	☐

ANXIETY / DEPRESSION

I am not anxious or depressed	☐
I am slightly anxious or depressed	☐
I am moderately anxious or depressed	☐
I am severely anxious or depressed	☐
I am extremely anxious or depressed	☐

We would like to know how good or bad your health is TODAY.

- This scale is numbered from 0 to 100.
- 100 means the best health you can imagine.
  0 means the worst health you can imagine.
- Mark an X on the scale to indicate how your health is TODAY.
- **Now, please write the number you marked on the scale in the box below.**

YOUR HEALTH TODAY = ☐

The best health you can imagine

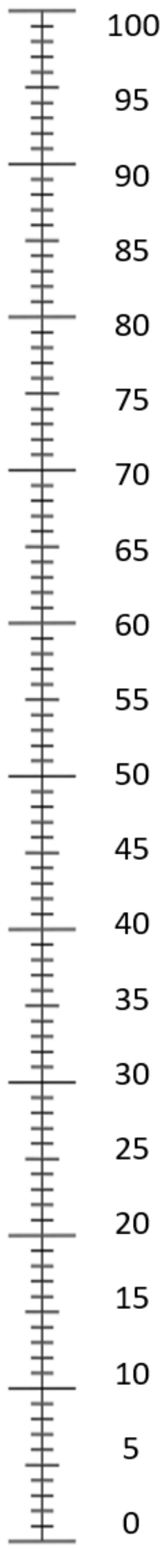

The worst health you can imagine

E. Falls Inventory (Baseline, 6M and 18M)

Please choose your answer by placing a tick (✔) in the box

1a. Have you had any falls including a slip or trip in which you lost your balance and landed on the floor or ground in the **past 6 months**?

☐ 1. Yes. ☐ 2. No

1b. **If yes**, how many times have you had this type of fall in **the last 6 months**?

☐ 1 ☐ 2 ☐ 3 ☐ 4 ☐ 5 or more

2. Have you experienced a fall **within the last 6 months**, resulting in an injury?

☐ 1. Yes
☐ 2. No

If Yes did you seek medical attention

☐ 1. Yes
☐ 2. No

F. Concern about falling (Baseline, 6M and 18M)

Now we would like to ask some questions about how concerned you are about the possibility of falling. Please reply thinking about how you usually do the activity. If you currently don't do the activity, please answer to show whether you think you would be concerned about falling IF you did the activity. For each of the following activities, please tick the box which is closest to your own opinion to show how concerned you are that you might fall if you did this activity.

Activity	Not at all concerned	Somewhat concerned	Fairly concerned	Very concerned
1. Getting dressed or undressed				
2. Taking a bath or shower				

3. Getting in or out of a chair				
4. Going up or down stairs				
5. Reaching for something above your head or on the ground				
6. Walking up or down a slope				
7. Going out to a social event (e.g. religious service, family gathering or club)				

This section to be conducted by a researcher

G. MEDICATIONS (Baseline, 6M, 12M and 18M)

A. Ask participants to hand in the prescribed medication form mailed to them prior to the assessment session (Medications CRF)
B. If not available, then support them in filling in a new medication form

H. Frailty Phenotype (Baseline, 6M and 18M)

1. Grip Strength

**Attempt 1**	
**Attempt 2**	
**Attempt 3**	
**Hand used**	**Left/Right**

2. Height (Baseline and 18M) and Weight (Baseline, 6M and 18M)

	**1st**	**2nd**	**3rd** (Only conducted if necessary)	**Final recorded value**
**Height**	**cms**	**cms**	**cms**	**cms**
**Weight**	**kg**	**kg**	**kg**	**kg**
**Shoes worn?**	**Yes/No**			

To be completed by the participant from this point

3. Weight loss

Over the last year have you lost 10lbs (4.5kg) or more in weight without intending to? Please circle Yes or No.

Yes	No

4. Exhaustion

1. Over the last month, how often did you feel that everything you did was an effort?

☐ Always	☐ Most of the time	☐ Sometimes	☐ Occasionally	☐ Never

2. Over the last month how often did you feel that you could not get going?

☐ Always	☐ Most of the time	☐ Sometimes	☐ Occasionally	☐ Never

I. Chronic Pain (Baseline, 6M and 18M)

The following questions will asked in the following format and you should give your answers by putting an “X” in one of the boxes.

NOTE: **1. If you put your “X” in the left-hand box, i.e.**

None	Mild	Moderate	Severe	Extreme
☒	☐	☐	☐	☐

Then you are indicating that you have no pain.

2. If you put your “X” in the right-hand box, i.e.

None	Mild	Moderate	Severe	Extreme
☐	☐	☐	☐	☒

Then you are indicating that your pain is extreme.

The following question concern the amount of pain you have experienced in your **hip**, **knee** or **ankle** joints. For each situation please enter the amount of pain experienced during the last week. (Please mark your answers with an ☒)

QUESTION: How much pain do you have?

1. Walking on a flat surface.

None	Mild	Moderate	Severe	Extreme
☐	☐	☐	☐	☐

2. Going up or down stairs.

None	Mild	Moderate	Severe	Extreme
☐	☐	☐	☐	☐

3. At night while in bed.

None	Mild	Moderate	Severe	Extreme
☐	☐	☐	☐	☐

4. Sitting or lying.

None	Mild	Moderate	Severe	Extreme
☐	☐	☐	☐	☐

5. Standing upright.

None	Mild	Moderate	Severe	Extreme
☐	☐	☐	☐	☐

J. Warwick-Edinburgh Mental Well-being Scale [WEMWBS]

(Baseline, 6M, 12M and 18M)

Below are some statements about feelings and thoughts.
Please tick (√) the answer that best describes your experience of each over the last 2 weeks

Question	None of the time	Rarely	Some of the time	Often	All of the time
1. I’ve been feeling optimistic about the future					
2. I’ve been feeling useful					
3. I’ve been feeling relaxed					
4. I’ve been feeling interested in other people					
5. I’ve had energy to spare					
6. I’ve been dealing with problems Well					
7. I’ve been thinking clearly					
8. I’ve been feeling good about Myself					
9. I’ve been feeling close to other people					
10. I’ve been feeling confident					
11. I’ve been to make up my own mind about things					
12. I’ve been feeling loved					
13. I’ve been interested in new things					
14. I’ve been feeling cheerful					

K. Capability [ICECAP-O] (Baseline, 6M, 12M and 18M)

ABOUT YOUR QUALITY OF LIFE

By placing a tick (√) in ONE box in EACH group below, please indicate which statement best describes your quality of life at the moment.

**1. Love and Friendship**		**Tick one box only in each section**
I can have all of the love and friendship that I want	☐	
I can have a lot of the love and friendship that I want	☐	
I can have a little of the love and friendship that I want	☐	
I cannot have any of the love and friendship that I want	☐	
**2. Thinking about the future**		
I can think about the future without any concern	☐	
I can think about the future with only a little concern	☐	
I can only think about the future with some concern	☐	
I can only think about the future with a lot of concern	☐	
**3. Doing things that make you feel valued**		
I am able to do all of the things that make me feel valued	☐	
I am able to do many of the things that make me feel valued	☐	
I am able to do a few of the things that make me feel valued	☐	
I am unable to do any of the things that make me feel valued	☐	
**4. Enjoyment and pleasure**		
I can have all of the enjoyment and pleasure that I want	☐	
I can have a lot of the enjoyment and pleasure that I want	☐	
I can have a little of the enjoyment and pleasure that I want	☐	
I cannot have any of the enjoyment and pleasure that I want	☐	
**5. Independence**		
I am able to be completely independent	☐	
I am able to be independent in many things	☐	
I am able to be independent in a few things	☐	
I am unable to be at all independent	☐	

L. Lubben Social Network Scale-6 (LSNS-6) (Baseline, 6M, 12M, 18M)

Instructions: Please circle the number that best describes your networks

**FAMILY:** ***Considering the people to whom you are related by birth, marriage, adoption etc…***
1. How many relatives do you see or hear from at least once a month?<br>0 = none 1 = one 2 = two 3 = three/ four 4 = five to eight 5 = nine or more
2. How many relatives do you feel at ease with that you can talk about private matters?<br>0 = none 1 = one 2 = two 3 = three/ four 4 = five to eight 5 = nine or more
3. How many relatives do you feel close to such that you could call on them for help?<br>0 = none 1 = one 2 = two 3 = three / four 4 = five to eight 5 = nine or more
**FRIENSHIPS:** ***Considering all of your friends including those who live in your neighbourhood***
4. How many of your friends do you see or hear from at least once a month?<br>0 = none 1 = one 2 = two 3 = three/ four 4 = five to eight 5 = nine or more
5. How many friends do you feel at ease with that you can talk about private matters?<br>0 = none 1 = one 2 = two 3 = three/ four 4 = five to eight 5 = nine or more
6. How many friends do you feel close to such that you could call on them for help?<br>0 = none 1 = one 2 = two 3 = three/ four 4 = five to eight 5 = nine or more

M.Revised UCLA loneliness scale (Baseline, 6M, 12M, 18M)

Instructions: Please indicate (√) how often each of the statements below is true for you.

Statement	Hardly Ever	Some of the time	Often
1. How often do you feel that you lack companionship?			
2. How often do you feel left out?			
3. How often do you feel isolated from others?			

N. Trips out of the house (Baseline, 6M, 12M, 18M)

In the past week (up to yesterday, not including today) how many times have you been out? By 'out' we mean a trip outside of your own home (not just to go to the garden, or balcony, or take the bins out).<br><br>Please choose your answer by placing a tick (✔) in the box					
☐ 0 trips /week	☐ 1-2 trips /week	☐ 3-4 trips /week	☐ 5-6 trips /week	☐ 7-8 trips week	☐ More than 9 trips/week

O. Medical History (Baseline)

We ask these questions to get a better picture of your overall health and quality of life. Again, all of your information will be kept strictly confidential.

1. Are you currently being treated for any of the following problems?

CHECK (✔) all that apply.

- ☐ a. Rheumatoid arthritis
- ☐ b. Diabetes
- ☐ c. Chronic Kidney Disease
- ☐ d. Osteoporosis
- ☐ e. Osteoarthritis
- ☐ f. Asthma
- ☐ g. COPD

Cardiovascular Disease

- ☐ h. Coronary Heart Disease
- ☐ i. High blood pressure (hypertension)
- ☐ j. Atrial Fibrillation
- ☐ k. Peripheral Arterial Disease.
- ☐ l. Stroke/Transient Ischaemic Attack (TIA or 'mini stroke')

Other conditions:

- ☐ m. Epilepsy
- ☐ n. Cancer
- ☐ o. Learning Disability

4. Have you been treated by a health care professional for any of the following?

   This question is in 2 parts: first, please think about the LAST 5 YEARS, then think about if you've EVER been treated for the condition.

   TICK all that apply.

Condition	Last 5 years	Ever	Never
a. Major depression	☐	☐	☐
b. Bipolar disorder	☐	☐	☐
c. Schizophrenia	☐	☐	☐
d. Psychosis	☐	☐	☐

P. Resource Use (Health and social care utilisation) (Baseline, 6M, 12M, 18M)

The following sections ask you about how much you have used different health services over the last 6 months. As you fill it in, please think about all the services you've used, for both your physical and mental health needs, as well as any social support needs. This will help us to understand the impact of our service upon your health and social care use. Please remember – all of these answers are completely confidential.

<u>**SECTION 1 HOSPITAL SERVICES**</u>

.

<u>Question 1</u>

Thinking over the **<u>last 6 months</u>**, what <u>hospital inpatient</u> services (treatment/care requiring an overnight stay in hospital) have you used? We have provided an example of how we would like you to complete this table. Please leave the table blank if the answer is zero.

**Number of times admitted to hospital in <u>last 6 months</u>**	**Total number of days in hospital** **(**If you were admitted more than once in last 6 months, please use the rows below to specify the number of nights spent in hospital for each time you were admitted**)**	**Date of visit (month/ year)**	**Was your stay in hospital planned ?**	**Please could you tell us the reason why you were in hospital**
Once	*5 days*	*March, 2021*	*no*	*Pneumonia required oxygen to help with breathing.*

<u>Question 2</u>

Thinking over the **<u>last 6 months</u>**, how many times have you had to go to **<u>accident & emergency (A&E)</u>** as a patient, but not had to stay overnight? We have provided an example of how we would like you to complete the table. Please leave the table blank if the answer is zero.

**Number A&E visits in <u>last 6 months</u>**	**Please specify the nature of the treatment received during the visit** e.g. type of clinic, investigative procedure, treatment received etc.
Once	*Injury of ankle requiring an x-ray and support bandage.*

<u>Question 3</u>

Thinking over the **<u>last 6 months</u>**, how many times have you attended **<u>hospital outpatient services</u>** as a patient? (All outpatients are so-called because they only stay in hospital for the length of their appointment – usually half-an-hour to an hour).

We have provided an example of how we would like you to complete the table. Please leave the table blank if the answer is zero.

**Number outpatient visits in <u>last 6 months</u>**	**Please specify the nature of the treatment received during the visit** e.g. clinic/department, investigative procedure, treatment received
Twice	*First visit: Breast clinic – received mammogram and biopsy.*
	Second visit: Arthritis clinic – received steroid injection

<u>Question 4</u>

Thinking over the **<u>last 6 months,</u>** <u>how many times have you attended</u> **<u>hospital as a day case patient? (</u>**A day case patient usually attends hospital for between a half and full day and usually has minor surgery and a period of time of supervised recovery. They do not stay overnight). We have provided an example of how we would like you to complete the table. Please leave the table blank if the answer is zero.

**Number daycase visits in <u>last 6 months</u>**	**Please specify the nature of the treatment received during the visit** e.g. clinic/department, investigative procedure, treatment received, day case surgery etc.
Twice	*First visit: Cataract surgery*
	Second visit: Varicose vein surgery

<u>SECTION 2: OTHER TYPES OF HEALTH CARE USE</u>

The next section asks you about any other contacts you have had with health professionals such as GP or practice nurses.

<u>Question 5</u>

Thinking about the **<u>last 6 months</u>**, please list any other contacts you have had with a health professional. We have provided an example of how we would like you to complete this table. Please leave blank if there were no contacts.

	**Number of contacts** (in last 6 months)	**Sector:** (please tick if this was private or on the NHS)		**Setting** (please tick if contact was at home or in a community setting i.e.GP surgery, NHS Drop-in centre)			**Average contact time (minutes) at each appointment or visit**
		**NHS/Social Care**	**Private**	**Community**	**Home**	**Over the phone or Internet**	
(Example) GP	*Two GP visits:*<br>*Visit 1*<br>*Visit 2*	√<br>√		√	√		*10 minutes*
GP							
Practice nurse							

Physiotherapist							
Cardiac Rehabilitation							
Pulmonary Rehabilitation							
Falls clinic							
Pain clinic							
District nurse							
Chiropodist or podiatrist							
Occupational therapist							
Osteopath							

Chiropractor							
Nutritionist							
Counsellor							
Acupuncturist							
Social care worker (provides home care services such as cooking meals, personal care or assisting with mobility)							
Other (please specify): ______________________							

Q6. Have you stayed in a Convalescent or Nursing Home during the last <u>6 months</u>?

Please tick ✔ one box only

☐ No   If **NO**, please go to **QUESTION 7**

☐ Yes   If **YES**, please give details below:

Number of days stayed in a convalescent or nursing home during the last **<u>6 months</u>** ☐☐☐

Did you pay for this convalescent/nursing home visit yourself?

☐ No   If **NO**, please tell us how it was paid for:

____

☐ Yes

Q7. Have relatives and/or friends helped you with tasks at home which you have had difficulty with or could not do in the last <u>6 months</u>?

Please tick ✔ one box only

☐ No   If **NO**, please go to **QUESTION 9**

☐ Yes   If **YES**, please give details below:

And you have had help from relatives and/or friends, typically how many hours per week? Hours per week = ☐☐☐

Q8: During the last 6 months, have relatives and/or friends stayed off work to help you?

Please tick ✔ one box only

☐ No   If **NO,** Please go to question 11

☐ Yes   If **YES**, please give details below:

How many days did they take off work in the last **<u>6 months</u>**? Days = ☐☐☐

Q9. Have you made changes to your home or special equipment (i.e. grab rails, ramps etc.) provided in last 6 months?
Please tick ✔ one box only

☐ No If **NO**, please go to **Q10 below**
☐ Yes If **YES**, please give details below:

Was this provided by social services?	Yes ☐	No ☐
If no, did you pay for it or contribute to cost?	Yes ☐	No ☐
Approximately how much?		

Q10. Do you care for a relative or friend on a regular basis? This means on a daily or weekly basis you help the person with their personal care needs such as eating or dressing, or with routine needs such as household chores, shopping, or business?
Please tick ✔ one box only

☐ No ☐ Yes

Q. Ageing Well Profile (Baseline, 6M and 18M)

In this section, we're interested in your well-being during the **last month**. Choose the box (✔) that best describes your feelings and thoughts about your health and wellbeing.

About your life in general…

**During the last month**:	**Not really true for me**		**Sort of true for me**		**Really true for me**
I have managed to sort out all my needs by myself.	1 ☐	2 ☐	3 ☐	4 ☐	5 ☐
In everyday life, I have not needed to rely on others.	1 ☐	2 ☐	3 ☐	4 ☐	5 ☐
I have been able to take good care of myself.	1 ☐	2 ☐	3 ☐	4 ☐	5 ☐
My life has given me a sense of accomplishment.	1 ☐	2 ☐	3 ☐	4 ☐	5 ☐
I have felt some good changes in myself.	1 ☐	2 ☐	3 ☐	4 ☐	5 ☐
My life has been really interesting.	1 ☐	2 ☐	3 ☐	4 ☐	5 ☐
I have felt that I have improved myself.	1 ☐	2 ☐	3 ☐	4 ☐	5 ☐

About your health and fitness…

**During the last month:**	**Not really true for me**		**Sort of true for me**		**Really true for me**
I have usually woken up fresh and rested	1 ☐	2 ☐	3 ☐	4 ☐	5 ☐
My body has felt strong enough to do what I wanted to do	1 ☐	2 ☐	3 ☐	4 ☐	5 ☐
On a day to day basis I have felt lively and healthy	1 ☐	2 ☐	3 ☐	4 ☐	5 ☐
I have not had many pains or much discomfort	1 ☐	2 ☐	3 ☐	4 ☐	5 ☐
My body has felt old and has limited what I could do	1 ☐	2 ☐	3 ☐	4 ☐	5 ☐
I have been able to keep going for longer than most people my age	1 ☐	2 ☐	3 ☐	4 ☐	5 ☐

**** Note for researcher: check correct completion of highlighted item***

About your feelings…

**During the last month**:	**Not really true for me**		**Sort of true for me**		**Really true for me**
I have had no doubts about who I am or my worth	1 ☐	2 ☐	3 ☐	4 ☐	5 ☐
I have rarely felt confused	1 ☐	2 ☐	3 ☐	4 ☐	5 ☐
I have not worried much about my life	1 ☐	2 ☐	3 ☐	4 ☐	5 ☐
I have been able to concentrate well when I wanted	1 ☐	2 ☐	3 ☐	4 ☐	5 ☐
I have had complete confidence in myself and my decisions	1 ☐	2 ☐	3 ☐	4 ☐	5 ☐
I have felt contented and happy with myself	1 ☐	2 ☐	3 ☐	4 ☐	5 ☐
I have been in a good mood more often than not	1 ☐	2 ☐	3 ☐	4 ☐	5 ☐

About your social life…

During the last month:	Not really true for me		Sort of true for me		Really true for me
	1	2	3	4	5
I have had plenty of people available to share my problems with	1 ☐	2 ☐	3 ☐	4 ☐	5 ☐
I have felt that people have enjoyed my company	1 ☐	2 ☐	3 ☐	4 ☐	5 ☐
I have rarely felt lonely or isolated	1 ☐	2 ☐	3 ☐	4 ☐	5 ☐
I have had lots of friends who I wanted to spend time with	1 ☐	2 ☐	3 ☐	4 ☐	5 ☐
I have spent a lot of my time with friends and acquaintances	1 ☐	2 ☐	3 ☐	4 ☐	5 ☐
My social life has been as good, as I would have liked	1 ☐	2 ☐	3 ☐	4 ☐	5 ☐
I have been able to give support and friendship to other people	1 ☐	2 ☐	3 ☐	4 ☐	5 ☐

P. Process evaluation (Baseline, 6M, 12M and 18M)

P.1 Muscle-strengthening or muscle-maintenance exercise

The UK Department of Health recommends that older adults should do **muscle-strengthening or maintenance** activities (exercise to improve or maintainmuscle strength, balance and flexibility) on at least two days a week. This can improve quality of life and reduce health risks. This means doing bouts of exercise (like squats, arm raises, or repeated lifting of weights) to work your arms, legs and other muscles in your body.
Please circle only one number that you feel best applies to you for each statement.

	Strongly disagree	Dis-Agree	Neither agree or disagree	Agree	Strongly agree
I normally do *less* muscle-strengthening exercise than recommended above.	1	2	3	4	5
Most weeks, I do the recommended amount of muscle-strengthening exercise.	1	2	3	4	5

Muscle-strengthening exercise questionnaire (0, 6, 12, 18 months)

In the questions below, "**Muscle strengthening exercise"** means doing exercises (like squats or repeated lifting of weights) to strengthen your arms, legs and other muscles in your body.

1. Doing muscle-strengthening exercises on at least two days a week is **very important to me**.

Strongly Disagree										Strongly Agree
0	1	2	3	4	5	6	7	8	9	10

2. **I am confident in my ability** to do two or more sessions of muscle-strengthening exercise each week.

Strongly Disagree										Strongly Agree
0	1	2	3	4	5	6	7	8	9	10

P.2 Physical activity –Perceived Benefits Scale

Please answer the following in relation to your own recent experiences of doing any physical activity or exercise over the last few months. If you have not done any such activity, feel free to “disagree” with the statements.
When we say **“Physical activity”** here, we mean any kind of physical activity or exercise that you have done, such as walking, gardening, , attending an exercise class, home exercising, or even dancing in the kitchen!

Please circle only one number that you feel best applies to you for each item

☐ Not applicable (I have not done any physical activity or exercise)

	**Strongly disagree**	**Dis-agree**	**Neither agree or disagree**	**Agree**	**Strongly Agree**
**Based on my recent experiences …**					
The physical activity that I have done has helped me to feel fit and healthy.	1	2	3	4	5
Doing physical activity has increased my overall energy levels.	1	2	3	4	5
Doing physical activity has improved my mood.	1	2	3	4	5
Doing physical activity has had benefits for my social life.	1	2	3	4	5

P.3 Activities in the local community

Research suggests that that engaging in organised activities in your local community is beneficial to your health and wellbeing.

When we say **"activities in your local community"** here, this might involve going to a social group, leisure activities like Bingo, doing classes (such as art, or computer classes,), church or faith-based activities, or volunteering activities that involve you getting out and about in the community.

☐ Not applicable (I have not done any local community activities)

Please circle only one number that you feel best applies to you for each item.

	Strongly disagree	Dis-Agree	Neither agree or disagree	Agree	Strongly agree
I do a lot of activity in my local community like the activities described above.	1	2	3	4	5
Most days of the week, I do an activity like the ones described above.	1	2	3	4	5
I hardly ever do this kind of activity	1	2	3	4	5

Please answer the following in relation to your own recent experiences of doing activities in your local community over the last few months. If you have not done any such exercise, feel free to "disagree" with the statements.

Please circle only one number that you feel best applies to you for each item

☐ Not applicable (I have not done any local community activities)

	Strongly disagree	Dis-agree	Neither agree or disagree	Agree	Strongly Agree
**Based on my recent experiences …**					
The activities that I have done in my local community have helped to keep me fit and healthy.	1	2	3	4	5

The activities that I have done in my local community have increased my overall energy levels.	1	2	3	4	5
The activities that I have done in my local community have improved my mood.	1	2	3	4	5
The activities that I have done in my local community have had benefits for my social life.	1	2	3	4	5

P.4 Your attitudes to physical activity and exercise

There are no right or wrong answers.

When we say **"Physical activity"** here, we mean any kind of physical activity or exercise that you have done, such as walking, gardening, attending an exercise class, home exercising, or even dancing in the kitchen!

Please *<u>circle</u>* *only one number that you feel best applies to you*

I am confident in my ability to do at least 30 minutes of physical activity on at least 5 days a week.

**Not confident at all**									**Extremely confident**	
0	1	2	3	4	5	6	7	8	9	10

Please *<u>circle</u>* *only one number that you feel best applies to you for each item.*

	**Strongly disagree**	**Dis-agree**	**Neither agree or disagree**	**Agree**	**Strongly Agree**
There are others in my life with whom I can be physically active.	1	2	3	4	5
I feel free to make my own decisions about physical activity.	1	2	3	4	5

There are people in my life who encourage me to be physically active.	1	2	3	4	5
In the last few months I have been doing physical activities that really interest me	1	2	3	4	5
I enjoy being physically active.	1	2	3	4	5
I feel pressured to be physically active.	5	4	3	2	1
Keeping up my current level of physical activity conflicts with other priorities in my life.	1	2	3	4	5
Keeping up my current level of physical activity over the next year would be a huge struggle.	1	2	3	4	5

In the questions below, **"Doing activities in my local community"** means going to a social group, leisure activities like Bingo, doing classes (such as art, or computer classes)church or faith-based activities, or volunteering activities that involve you getting out and about in the community.

I am confident in my ability to engage in activities in my local community at least 3 times each week.

Strongly Disagree										Strongly Agree
0	1	2	3	4	5	6	7	8	9	10

Please circle only one number that you feel best applies to you for each item.

☐ Not applicable (I have not done any activities in my local community)

	Strongly disagree	Dis-agree	Neither agree or disagree	Agree	Strongly agree
There are others in my life with whom I can do activities in the local community.	1	2	3	4	5
I feel free to make my own decisions about activities that I do in my local community.	1	2	3	4	5
There are people in my life who encourage me to do activities in my local community.	1	2	3	4	5
In the last few months I have been doing activities in my local community that really interest me	1	2	3	4	5
I enjoy doing activities in my local community.	1	2	3	4	5
I feel pressured to do activities in my local community.	5	4	3	2	1
Keeping up my current level of activities in my local community conflicts with other priorities in my life.	1	2	3	4	5
Keeping up my current level of activities in my local community over the next year would be a huge struggle.	1	2	3	4	5

P. 5 Evaluation of local area surroundings (baseline, 18 months)

In this section, we're going to ask about your local area and how much you agree with various statements. Please think about the area around your home within a **10-15 minute walk, in any direction**, and choose the answer that best applies to you and your local area.

1. It is easy and pleasant to walk in my local area

1	2	3	4
strongly disagree	somewhat disagree	somewhat agree	strongly agree

2. The streets in my local area are hilly, making it difficult for me to walk to places I want to visit

1	2	3	4
strongly disagree	somewhat disagree	somewhat agree	strongly agree

3. The pavements in my local area are well-maintained (paved, even, not a lot of cracks)

1	2	3	4
strongly disagree	somewhat disagree	somewhat agree	strongly agree

4. The traffic on <u>nearby</u> streets makes it difficult or unpleasant to walk in my local area

1	2	3	4
strongly disagree	somewhat disagree	somewhat agree	strongly agree

5. At major road junctions in my local area, there are islands in the middle of the road where pedestrians can safely stop after crossing half way

1	2	3	4
strongly disagree	somewhat disagree	somewhat agree	strongly agree

6. There are dropped curbs that go from pavement level to road level in my local area

1	2	3	4
strongly disagree	somewhat disagree	somewhat agree	strongly agree

7. How safe would you feel walking alone in this area **after dark**?

1	2	3	4	5
Very safe	Fairly safe	A bit unsafe.	Very unsafe	Never go out alone

8. How safe would you feel walking alone in this area **in the daytime**?

1	2	3	4	5
Very safe	Fairly safe	A bit unsafe.	Very unsafe	Never go out alone

9. How much does poor availability of benches for stopping and resting restrict you from getting out and about in the local area eg. walking to local shops or visiting neighbours?

Not at all/hardly at all*	A little bit	Moderately so	A lot
1	2	3	4

*If you feel there are enough benches, score as 1

10. Taking everything into account, what do you think of your local area as a place to live (for you)?

A very good place to live	A fairly good place to live	Neither good nor bad	A fairly bad place to live	A very bad place to live
1	2	3	4	5

Thank you!

That's it – thank you very much for completing the measurement session! We hope that you have enjoyed thinking about some of these ideas.
