# Supplementary material for: A peer-volunteer led active ageing programme to prevent decline in physical function in older people at risk of mobility disability (Active, Connected, Engaged [ACE]): study protocol for a randomised controlled trial
Source: Trials. 2023 Nov 29;24:772. doi: 10.1186/s13063-023-07758-3 (PMC10687817; doi:10.1186/s13063-023-07758-3)
Supplement: Supplementary file 4 — Additional file 4. ACE original ethical approval dated 11th August 2021. [file 13063_2023_7758_MOESM4_ESM.pdf]

NHS
Health Research
Authority

London - Surrey Research Ethics Committee
Nottingham Centre
The Old Chapel
Royal Standard Place
Nottingham
NG1 6FS

<u>Please note</u>: This is the favourable opinion of the REC only and does not allow you to start your study at NHS sites in England until you receive HRA Approval

11 August 2021

Dr Afroditi Stathi
Associate Professor/Reader in Active Ageing and Health
University of Birmingham
School of Sport, Exercise and Rehabilitation Sciences
University of Birmingham
Edgbaston, Birmingham
B15 2TT

Dear Dr Stathi

**Study title:**	**A multi-centre randomised controlled trial of a peer-volunteer led active ageing programme to prevent decline in physical function in older people at risk of mobility disability. The ACE (Active, Connected,Engaged) study**
**REC reference:**	**21/LO/0433**
**IRAS project ID:**	**290332**

Thank you responding to the Research Ethics Committee's (REC) request for further information on the above research and submitting revised documentation.

The further information has been considered on behalf of the Committee by the Chair.

Confirmation of ethical opinion

On behalf of the Committee, I am pleased to confirm a favourable ethical opinion for the above research on the basis described in the application form, protocol and supporting documentation as revised, subject to the conditions specified below.

Good practice principles and responsibilities

The UK Policy Framework for Health and Social Care Research sets out principles of good practice in the management and conduct of health and social care research. It also outlines the responsibilities of individuals and organisations, including those related to the four elements of research transparency:

1. registering research studies
2. reporting results
3. informing participants
4. sharing study data and tissue

Conditions of the favourable opinion

The REC favourable opinion is subject to the following conditions being met prior to the start of the study.

Confirmation of Capacity and Capability (in England, Northern Ireland and Wales) or NHS management permission (in Scotland) should be sought from all NHS organisations involved in the study in accordance with NHS research governance arrangements. Each NHS organisation must confirm through the signing of agreements and/or other documents that it has given permission for the research to proceed (except where explicitly specified otherwise).

Guidance on applying for HRA and HCRW Approval (England and Wales)/ NHS permission for research is available in the Integrated Research Application System.

For non-NHS sites, site management permission should be obtained in accordance with the procedures of the relevant host organisation.

Sponsors are not required to notify the Committee of management permissions from host organisations

Registration of Clinical Trials

All research should be registered in a publicly accessible database and we expect all researchers, research sponsors and others to meet this fundamental best practice standard.

It is a condition of the REC favourable opinion that **all clinical trials are registered** on a publicly accessible database within six weeks of recruiting the first research participant. For this purpose, 'clinical trials' are defined as the first four project categories in IRAS project filter question 2. Failure to register a clinical trial is a breach of these approval conditions, unless a deferral has been agreed by or on behalf of the Research Ethics Committee (see here for more information on requesting a deferral:

https://www.hra.nhs.uk/planning-and-improving-research/research-planning/research-registration-research-project-identifiers/

If you have not already included registration details in your IRAS application form, you should notify the REC of the registration details as soon as possible.

Further guidance on registration is available at:
https://www.hra.nhs.uk/planning-and-improving-research/research-planning/transparency-responsibilities/

Publication of Your Research Summary

We will publish your research summary for the above study on the research summaries section of our website, together with your contact details, no earlier than three months from the date of this favourable opinion letter.

Should you wish to provide a substitute contact point, make a request to defer, or require further information, please visit:
https://www.hra.nhs.uk/planning-and-improving-research/application-summaries/research-summaries/

N.B. If your study is related to COVID-19 we will aim to publish your research summary within 3 days rather than three months.

During this public health emergency, it is vital that everyone can promptly identify all relevant research related to COVID-19 that is taking place globally. If you haven't already done so, please register your study on a public registry as soon as possible and provide the REC with the registration detail, which will be posted alongside other information relating to your project. We are also asking sponsors not to request deferral of publication of research summary for any projects relating to COVID-19. In addition, to facilitate finding and extracting studies related to COVID-19 from public databases, please enter the WHO official acronym for the coronavirus disease (COVID-19) in the full title of your study. Approved COVID-19 studies can be found at:
https://www.hra.nhs.uk/covid-19-research/approved-covid-19-research/

It is the responsibility of the sponsor to ensure that all the conditions are complied with before the start of the study or its initiation at a particular site (as applicable).

After ethical review: Reporting requirements

The attached document "After ethical review – guidance for researchers" gives detailed guidance on reporting requirements for studies with a favourable opinion, including:

- Notifying substantial amendments
- Adding new sites and investigators
- Notification of serious breaches of the protocol
- Progress and safety reports
- Notifying the end of the study, including early termination of the study
- Final report
- Reporting results

The latest guidance on these topics can be found at https://www.hra.nhs.uk/approvals-amendments/managing-your-approval/.

Ethical review of research sites

NHS/HSC sites

The favourable opinion applies to all NHS/HSC sites taking part in the study, subject to confirmation of Capacity and Capability (in England, Northern Ireland and Wales) or management permission (in Scotland) being obtained from the NHS/HSC R&D office prior to the start of the study (see "Conditions of the favourable opinion" below).

Non-NHS/HSC sites

I am pleased to confirm that the favourable opinion applies to any non-NHS/HSC sites listed in the application, subject to site management permission being obtained prior to the start of the study at the site.

Approved documents

The final list of documents reviewed and approved by the Committee is as follows:

Document	*Version*	*Date*
Copies of materials calling attention of potential participants to the research [ACE Press Release]	1	01 March 2021
Copies of materials calling attention of potential participants to the research [ACE Participant Mailed Reply Form]	1	01 March 2021
Covering letter on headed paper	1	13 May 2021
Covering letter on headed paper [ACE Cover letter]	1.1	27 July 2021
Evidence of Sponsor insurance or indemnity (non NHS Sponsors only)	1	13 May 2021
GP/consultant information sheets or letters [ACE GP Info Sheet]	1	01 March 2021
GP/consultant information sheets or letters [ACE GP Invitation Letter]	1	01 March 2021
Interview schedules or topic guides for participants [Interview Guide ACE Participants]	1	01 March 2021
Interview schedules or topic guides for participants [Interview Guide ACE Volunteers]	1	01 March 2021
Interview schedules or topic guides for participants [Interview Guide ACE Managers]	1	01 March 2021
IRAS Application Form [IRAS_Form_18052021]		18 May 2021
Letter from funder	1	01 March 2021
Letter from sponsor	1	13 May 2021
Letter from statistician	1	15 March 2021
Letters of invitation to participant [ACE Participant Letter GP]	1	01 March 2021
Letters of invitation to participant [ACE Stakeholder Invitation Letter]	1	01 March 2021
Non-validated questionnaire [ACE Telephone Screening Phone Script]	1	01 March 2021

Non-validated questionnaire [ACE Time and Travel Diary]	1	01 March 2021
Non-validated questionnaire [RVS Volunteer Info Form Tracked changes]	1.1	21 June 2021
Non-validated questionnaire [Telephone Screening Form Tracked Changes]	1.1	21 June 2021
Other [Response to REC feedback]	1	21 June 2021
Participant consent form [Participant Consent Form Tracked changes]	1.1	21 June 2021
Participant consent form [Volunteer Consent Form Tracked changes]	1.1	21 June 2021
Participant consent form [Volunteer Manager Consent Form Tracked changes]	1.1	21 June 2021
Participant consent form [Stakeholder Consent Form Tracked Changes]	1.1	21 June 2021
Participant consent form [Participant Consent Form]	1.1	21 June 2021
Participant consent form [Volunteer Consent Form]	1.1	21 June 2021
Participant consent form [Volunteer Manager Consent Form]	1.1	21 June 2021
Participant consent form [Stakeholder Consent Form]	1.1	21 June 2021
Participant consent form [ACE Participant Consent Form V1.1 210621]	V1.1	21 June 2021
Participant information sheet (PIS) [Participant Information Sheet Tracked Changes]	1.1	21 June 2021
Participant information sheet (PIS) [ACE Volunteer Information Sheet Tracked changes]	1.1	21 June 2021
Participant information sheet (PIS) [Participant Info Sheet]	1.1	21 June 2021
Participant information sheet (PIS) [Volunteer Information Sheet]	1.1	21 June 2021
Research protocol or project proposal [ACE study Protocol]	1	01 March 2021
Summary CV for Chief Investigator (CI) [Summary CV for CI]	1	01 March 2021
Summary, synopsis or diagram (flowchart) of protocol in non technical language	1	01 March 2021
Validated questionnaire [ACE CRF Medications]	1	01 March 2021
Validated questionnaire [Participant CRF Tracked Changes]	1.1	21 June 2021
Validated questionnaire [Volunteer CRF Tracked changes]	1.1	21 June 2021

Statement of compliance

The Committee is constituted in accordance with the Governance Arrangements for Research Ethics Committees and complies fully with the Standard Operating Procedures for Research Ethics Committees in the UK.

User Feedback

The Health Research Authority is continually striving to provide a high quality service to all applicants and sponsors. You are invited to give your view of the service you have received and the application procedure. If you wish to make your views known please use the feedback form available on the HRA website:
http://www.hra.nhs.uk/about-the-hra/governance/quality-assurance/

HRA Learning

We are pleased to welcome researchers and research staff to our HRA Learning Events and online learning opportunities– see details at:
https://www.hra.nhs.uk/planning-and-improving-research/learning/

**IRAS project ID: 290332**	**Please quote this number on all correspondence**

With the Committee’s best wishes for the success of this project.

Yours sincerely

PP.

Hayleigh Morris
Approvals Administrator

Mrs Chrissie Lawson
Chair

Email:surrey.rec@hra.nhs.uk

Enclosures:	“After ethical review – guidance for researchers”
Copy to:	Dr Birgit Whitman

Lead Nation - England: approvals@hra.nhs.uk
